# Supplementary material for: Cooperative allostery and structural dynamics of streptavidin at cryogenic- and ambient-temperature
Source: Commun Biol. 2022 Jan 20;5:73. doi: 10.1038/s42003-021-02903-7 (PMC8776744; doi:10.1038/s42003-021-02903-7)
Supplement: Supplementary file 2 — Supplementary Information [file 42003_2021_2903_MOESM2_ESM.docx]

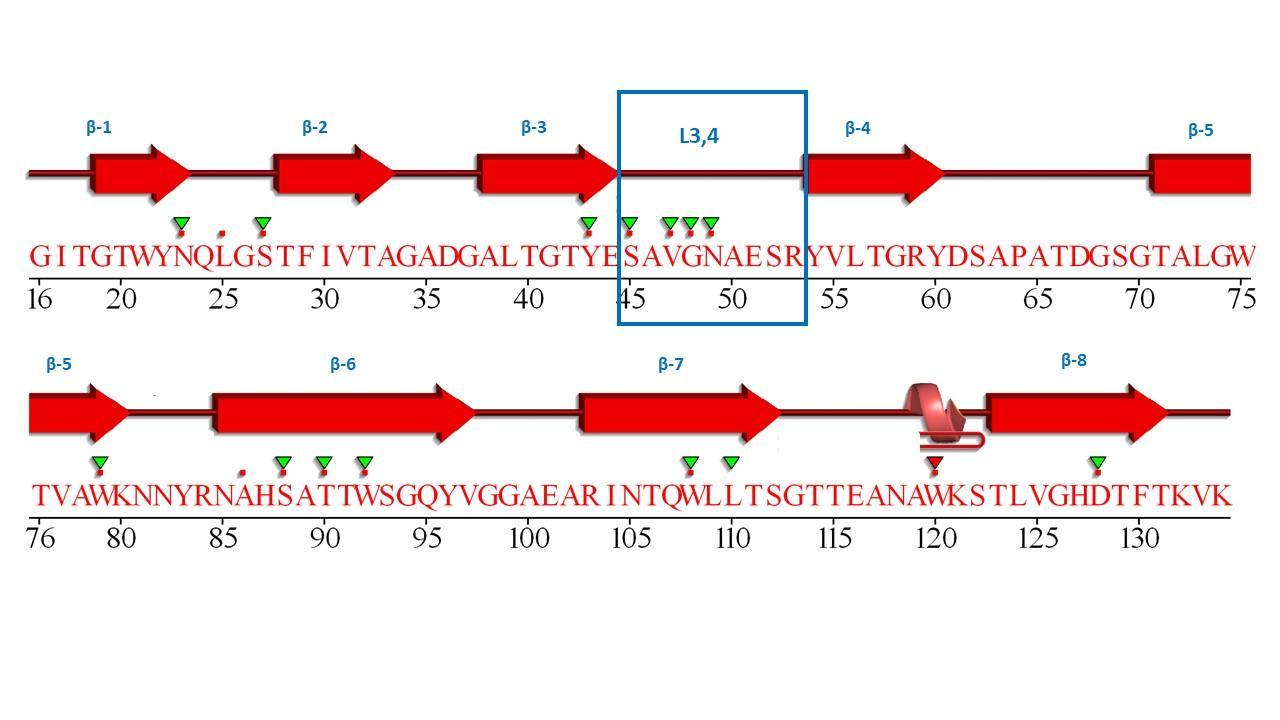


**Supplementary Fig. 1: Secondary structure and sequence of streptavidin.** The arrows indicate ß-strands, and the helix shape represents α-helice. Flat U turns shapes represent flexible loop. Loop 3/4 marked with a square and labeled as L3/4. Red dots represent residues in contact with ligand (selenobiotin), while inverted triangles that are colored in green and red represent functional residues of repeats. This figure was created using the PDBsum server and modified [70].


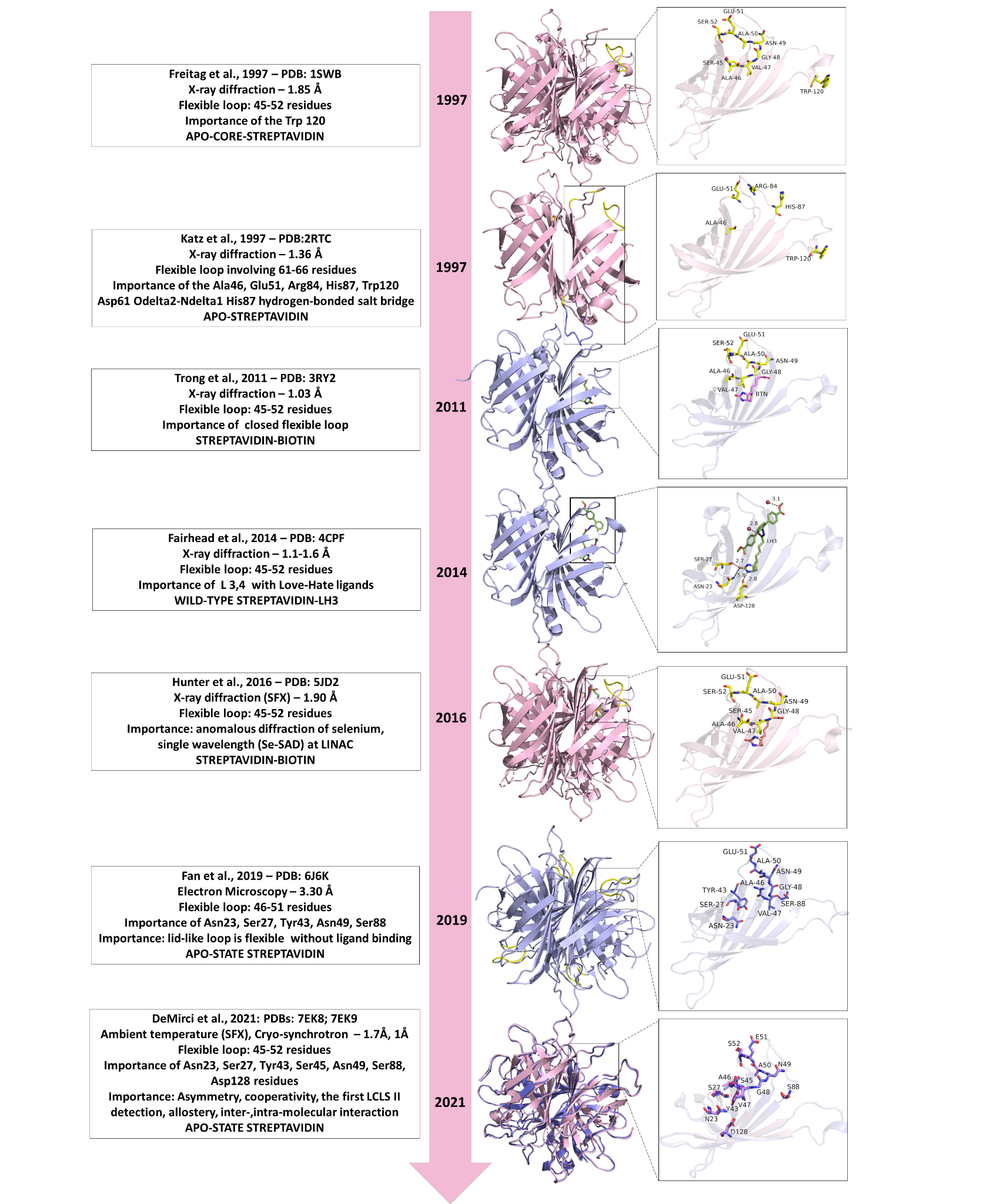


**Supplementary Fig. 2:** Chronological partial-bibliography of the apo- and holo-streptavidin structures. **1997:** It was emphasized on the 45-52 inter-residual loop (L3/4) concept and the importance of the Trp120 residue in apo-streptavidin (PDB_ID: 1SWB). **1997**: Simultaneous X-ray diffraction model. Its resolution is higher than the previous model. It was emphasized the importance of the loop 61-66 rather than L3/4 as well as of His87, Ala46, Glu51, Trp120and Arg-84. Additionally, it was indicated the importance of the salt bridge between Asp61 and His87 (PDB_ID: 2RTC). **2011:** Relatively high-resolution streptavidin-biotin model compared to the previous structures. Attention had been drawn to the concept of a "closed-flexible" L3/4 residues (PDB_ID: 3RY2). **2014:** In order to understand the protein plasticity, conflicted ligands were used instead of biotin. The importance of Ser-45 in L3/4 cycle is emphasized with the LH3-bound wild-type streptavidin structure (PDB_ID: 4CPF) **2016**: It was indicated the first SFX streptavidin-biotin model that has been studied with ambient-temperature X-FEL. The L3/4 conformation is pointed out, and for the first time, selenobiotinyl-streptavidin structure demonstrated by using phases obtained by the anomalous diffraction of selenium measured at a single wavelength (Se-SAD) at the Linac Coherent Light Source (PDB_ID: 5JD2). **2019**: It was demonstrated the first apo-state streptavidin model powered by Cryo-EM. Current work refers to the concept of a "lid” like loop without ligand-binding, paying attention to loop conformation between 51-56 residues and its interaction with active residues (PDB_ID: 6J6K). **2021:** This work emphasizes the first models to be extensively investigated apo-core streptavidin powered by radiation-damage free SFX (Apo-SFX, PDB: 7EK8) as well as high-resolution cryo-synchrotron (Apo-Cryo, PDB: 7EK9). The L3/4 conformation has been given importance. First LCLS II study, involving inter- and intra- monomeric examination, compares the interaction of its active residues with the previous study (PDB_ID: 5JD2).


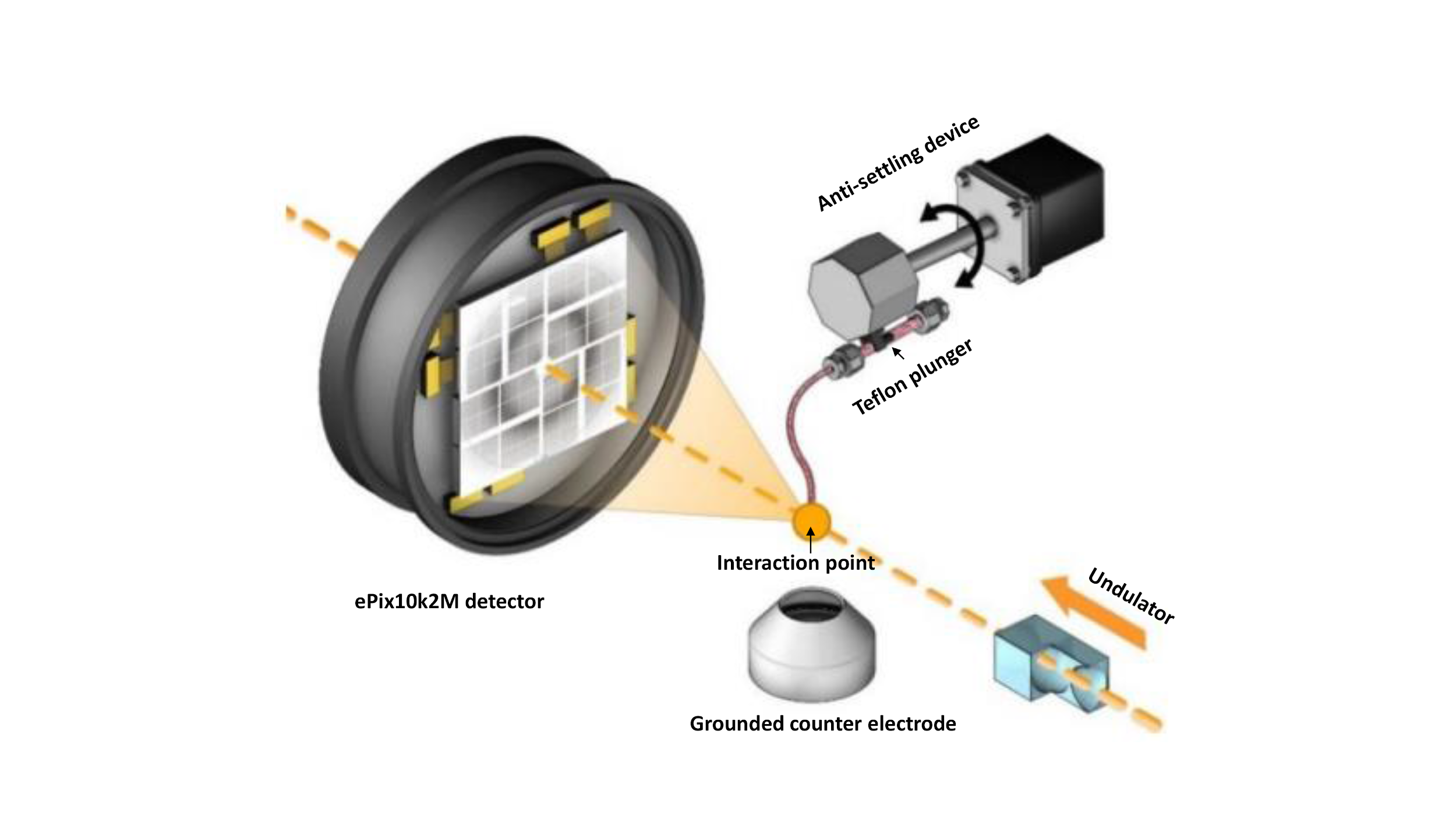


**Supplementary Fig. 3: Diagram of the MESH injector setup at the XFEL.** Protein microcrystals are injected by this injector. The sample reservoir has a Teflon plunger (indicated by arrow). This reservoir is attached to an anti-settling device rotating at an angle to prevent crystal settling of proteins and keep them homogenized. The protein crystals and the LCLS pulses interacted at the point indicated by the arrow. After the X-ray beam hits these crystals at the interaction point (indicated by an arrow), diffraction data is collected by the ePix10k2M camera.


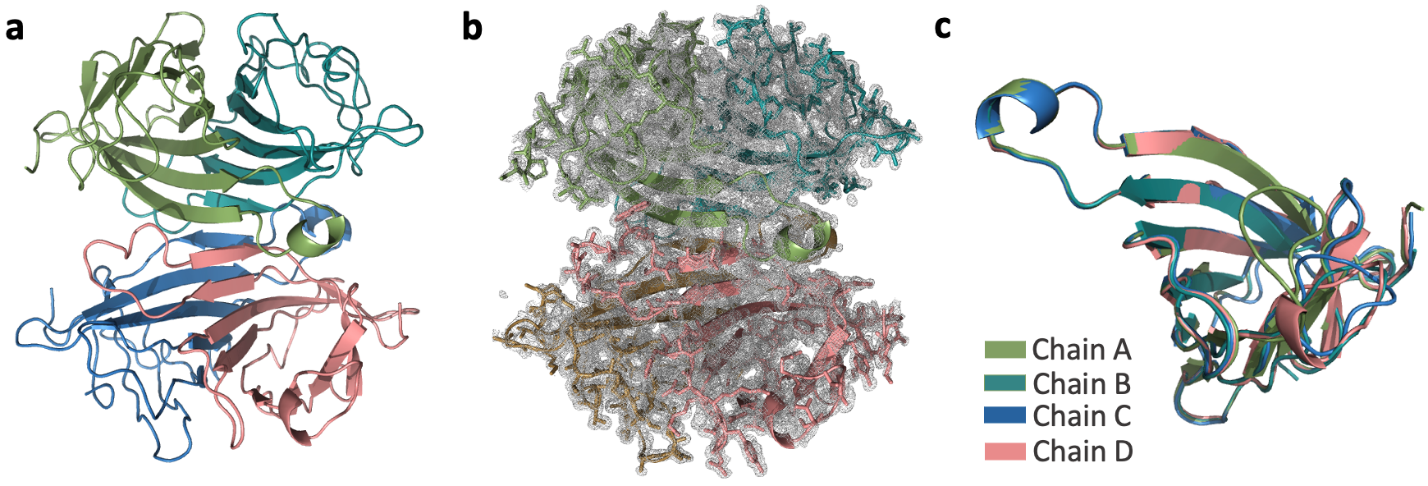


**Supplementary Fig. 4: Synchrotron structure of streptavidin (Apo-Cryo).** **(a)** The apo structure of streptavidin is colored based on each chain. **(b)** 2*F*o-*F*c simulated annealing-omit map at 1 sigma level is colored in gray. **(c)** Each chain of streptavidin is superposed with an overall RMSD of 0.136 Å.


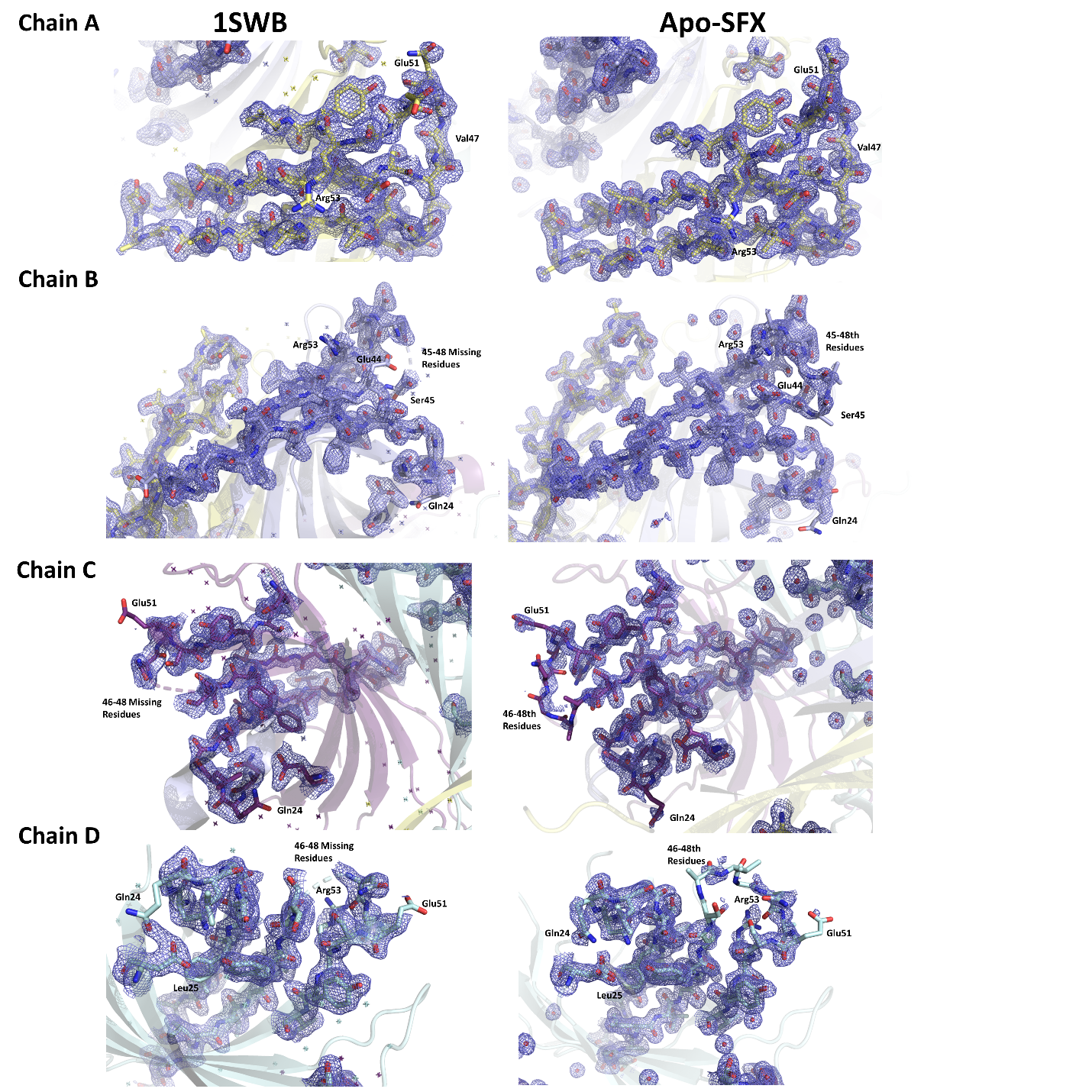


**Supplementary Fig. 5: Electron density map comparison of binding site residues of the 1SWB and Apo-SFX structure of streptavidin.** 2*F*o-*F*c simulated electron density map is colored in slate. Chain colors are presented as described before. 1SWB structure has missing residues between 46-48th position in chain B, C and D. Moreover, Gln24, Lue25, Val47, Glu51, Arg53 were observed with better electron density for Apo-SFX structure.


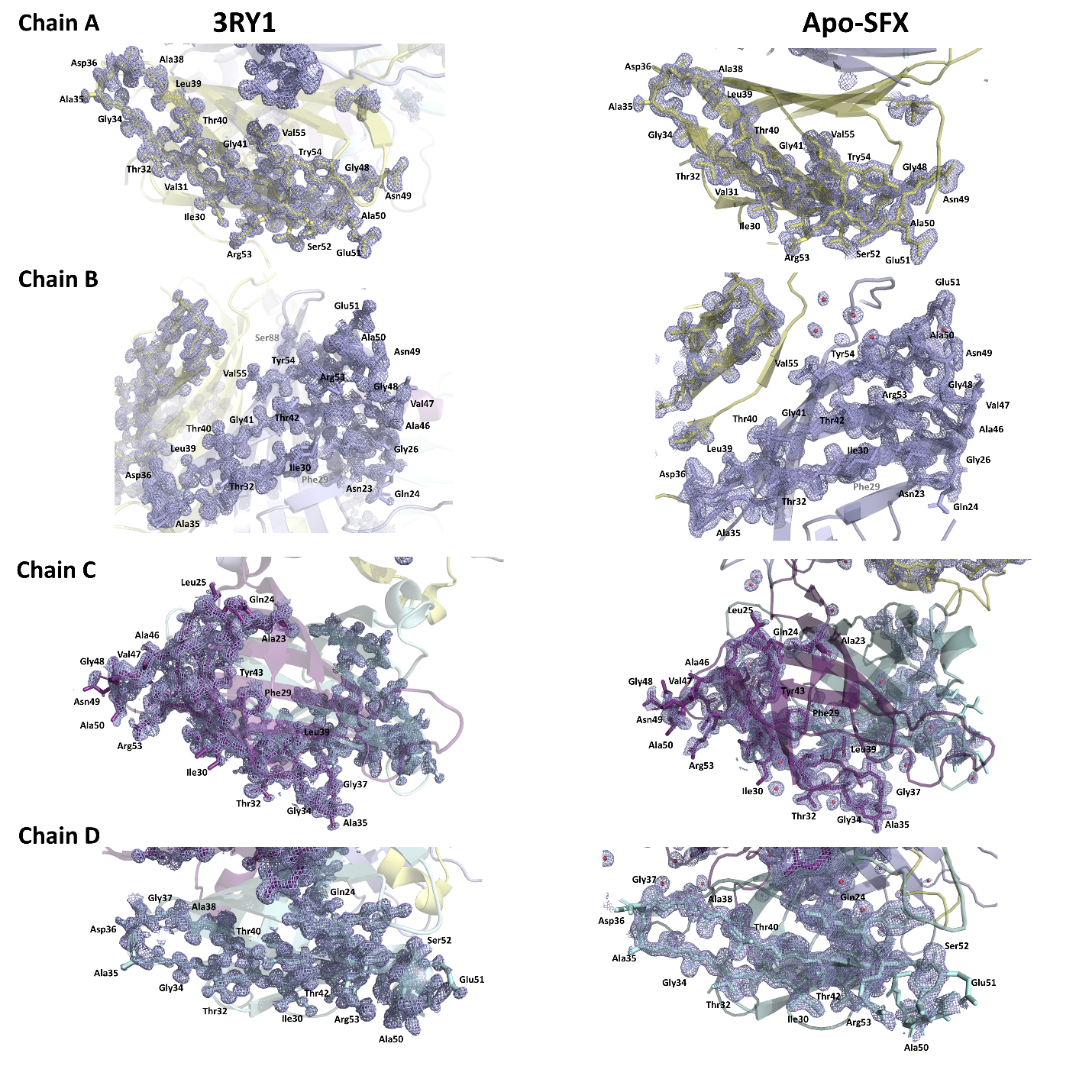


**Supplementary Fig. 6: Electron density map comparison of binding site residues of the 3RY1 and Apo-SFX structure of streptavidin.** 2*F*o-*F*c simulated electron density map is colored in slate. Chain colors are presented as described before. For Apo-SFX structure, chains A and B were observed with enhanced and continuous electron density for those binding residues compared to 3RY1 structure. However, in chain C and D, Apo-SFX structure was observed with better electron density at the beginning and end of the binding site residues such as Asn23 or Glu51, while L3/4 was identified with better electron density at 3RY1 structure.

**
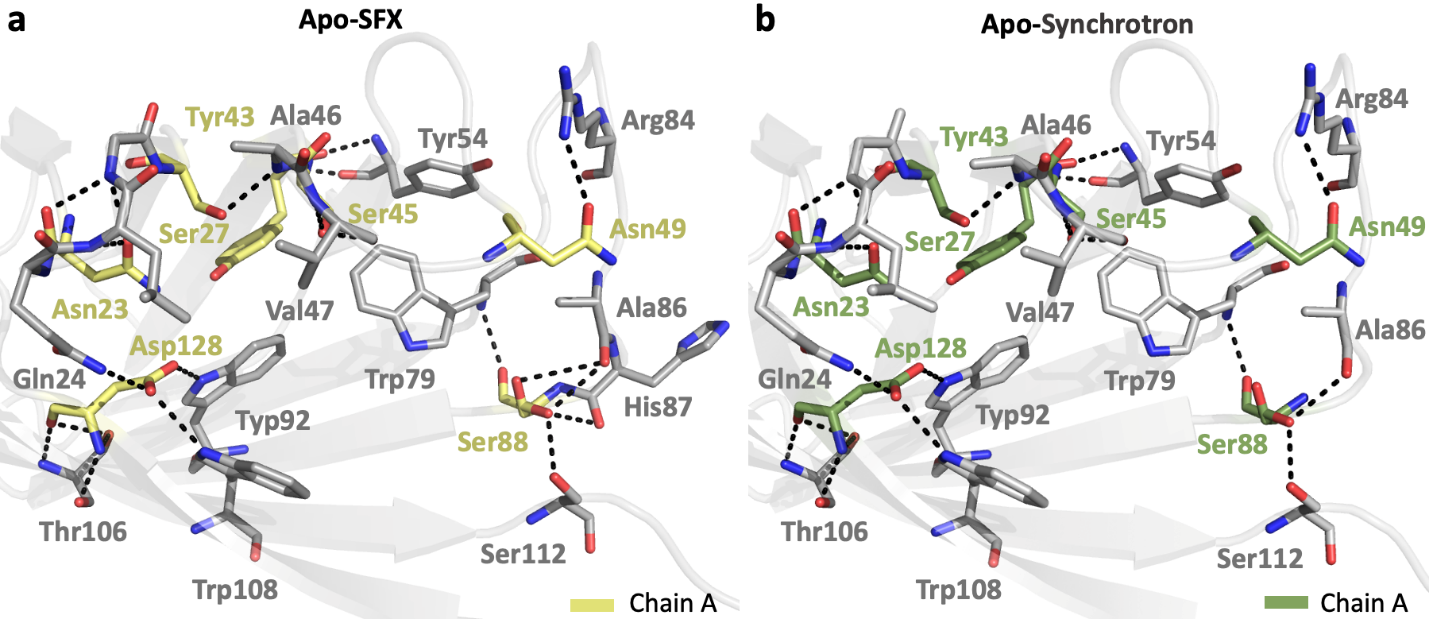
**

**Supplementary Fig. 7: Hydrogen bond interactions in the binding pocket. (a)** The interactions within 1-3.5 Å are represented by dashed lines and active residues in chain A of SFX structure are colored in pale yellow while flank cavity residues are colored in gray. **(b)** The interactions within 1-3.5 Å are represented by dashed lines and active residues in chain A of synchrotron structure are colored in green while flank cavity residues are colored in gray.


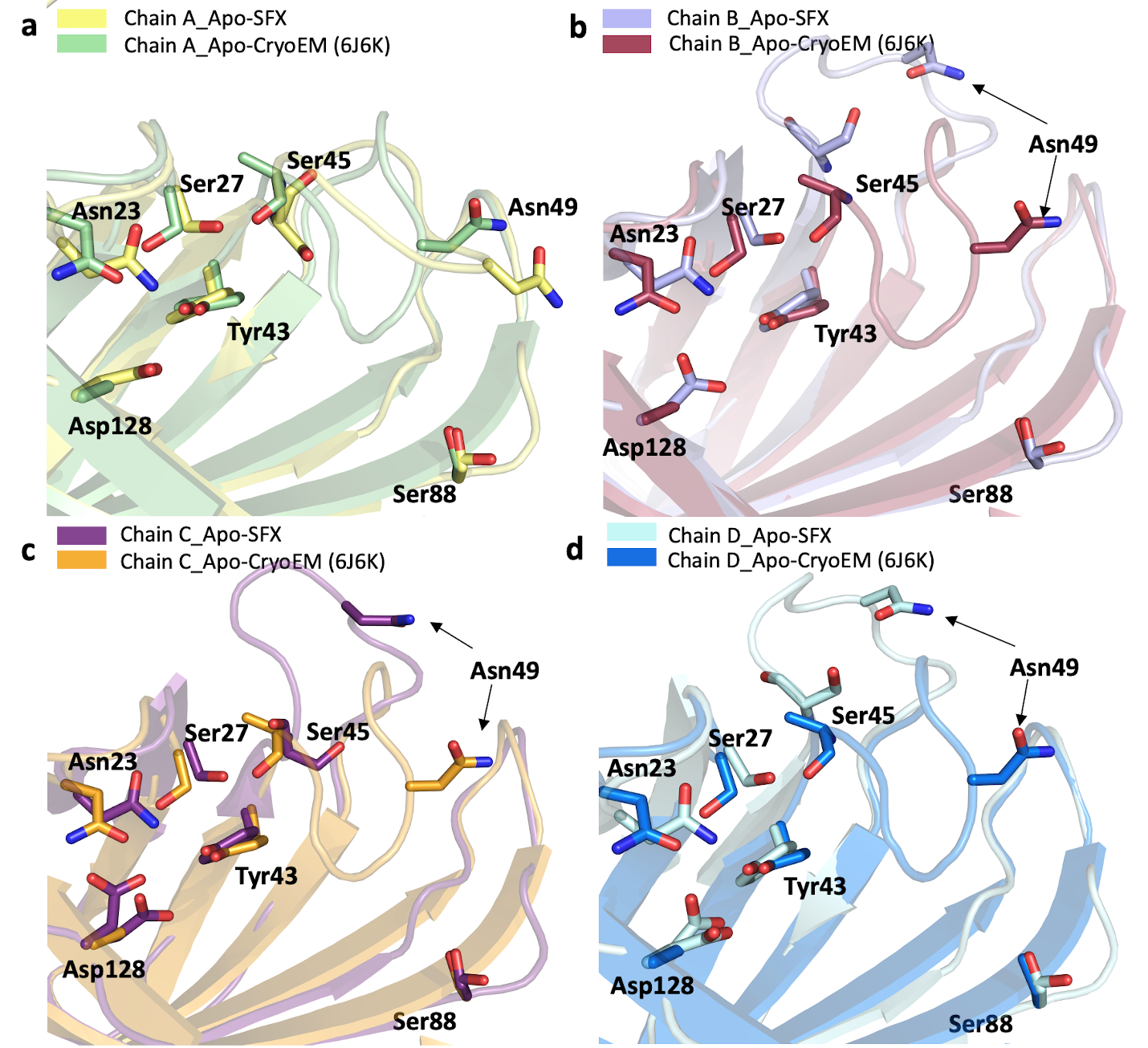


**Supplementary Fig. 8: Biotin binding site of Apo-SFX structure compared with** **Apo-CryoEM (PDB ID: 6J6K).** Chain A-D of Apo-SFX is superposed with streptavidin structure (PDB ID:6J6K) in panel a-d, respectively (Supp Table 2) Conformational changes are observed on the L3/4 region where the residue 49 is indicated with black arrow for each chain compared to apo-state streptavidin (PDB ID:6J6K).

**
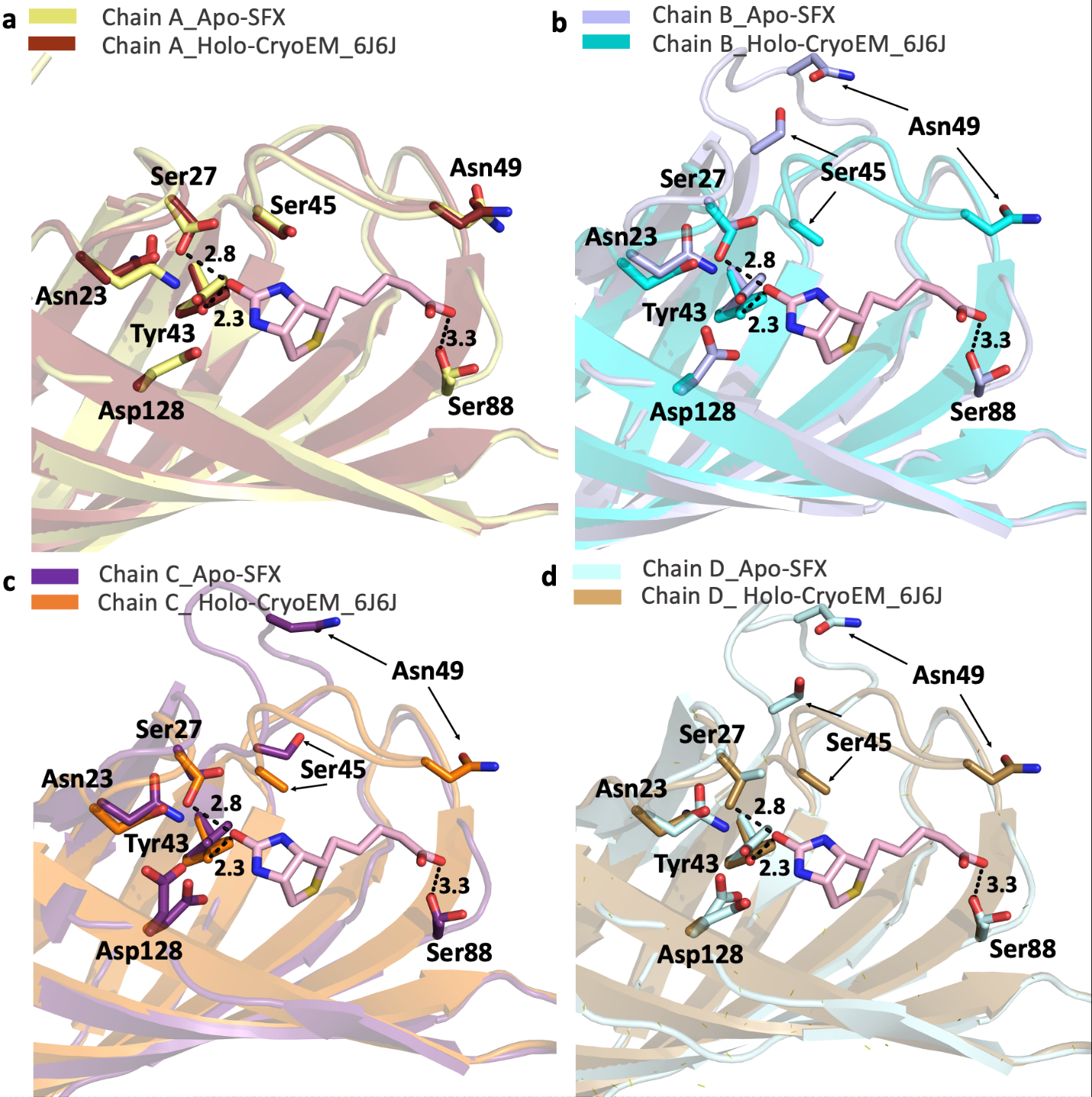
**

**Supplementary Fig. 9: Superposition of our Apo-SFX structure and Holo-CryoEM structure of streptavidin in complex with biotin (PDB ID: 6J6J). a)** Superposition of Chain A of Apo-SFX and biotin-bound (PDB ID: 6J6J) streptavidin structures with a RMSD of 0.38 Å. **b)** Superposition of Chain B of two streptavidin structures has an RMSD of 0. 41 Å. **c)** Superposition of Chain C of two structures with a RMSD of 0. 41 Å. **d)** Superposition of Chain D of two structures with a RMSD of 0. 37 Å.


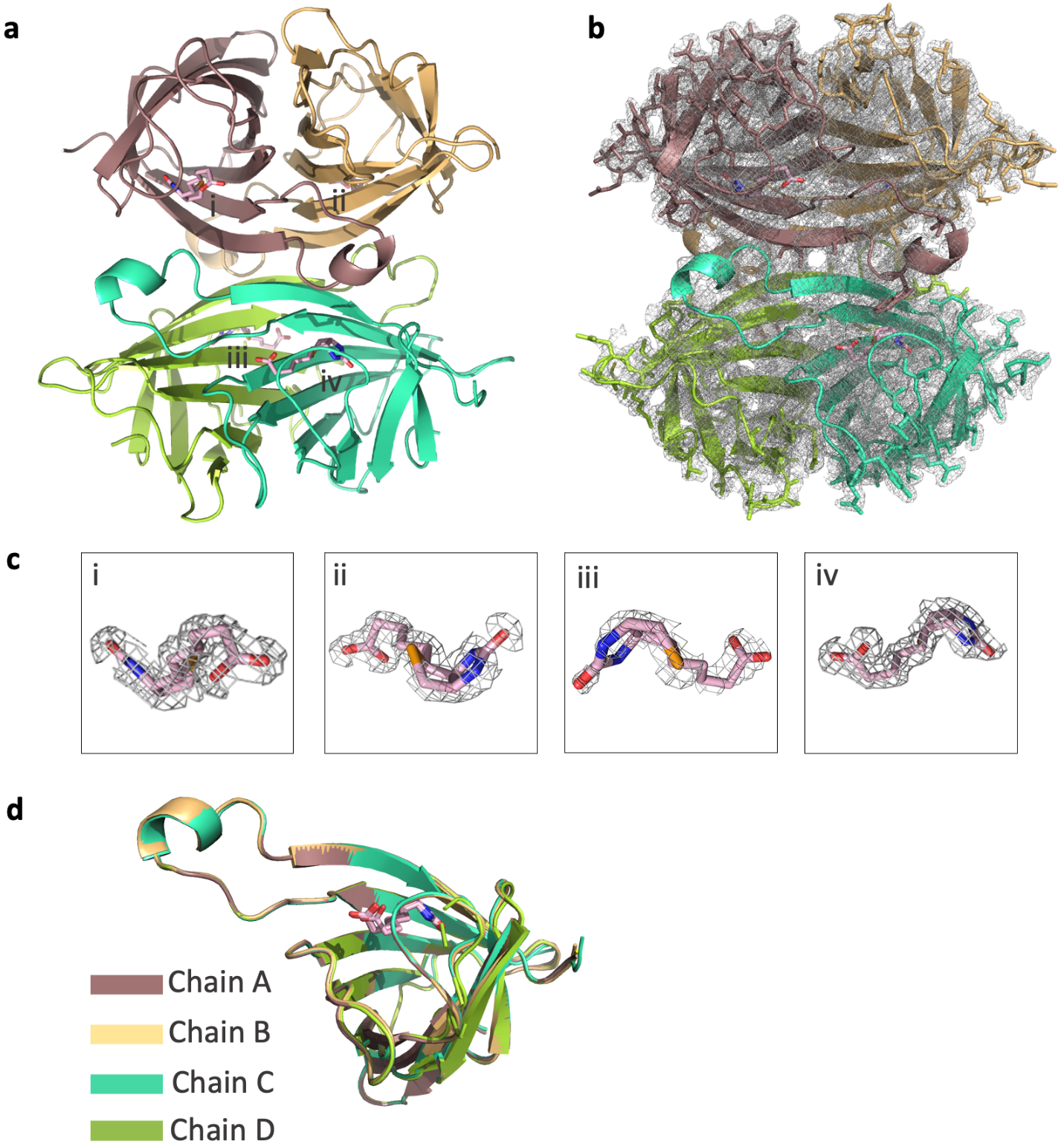


**Supplementary Fig. 10: Holo-SFX structure bound with selenobiotin (PDB ID: 5JD2). (a)** Holo-SFX structure is colored based on chain. **(b)** 2*F*o-*F*c simulated annealing-omit map at 1 sigma level is colored in gray. **(c)** 2*F*o-*F*c simulated annealing-omit map of four selonobiotins (light pink) at 1 sigma level are colored in gray. **(d)** Each chain of streptavidin is superposed with an overall RMSD of 0.13 Å.


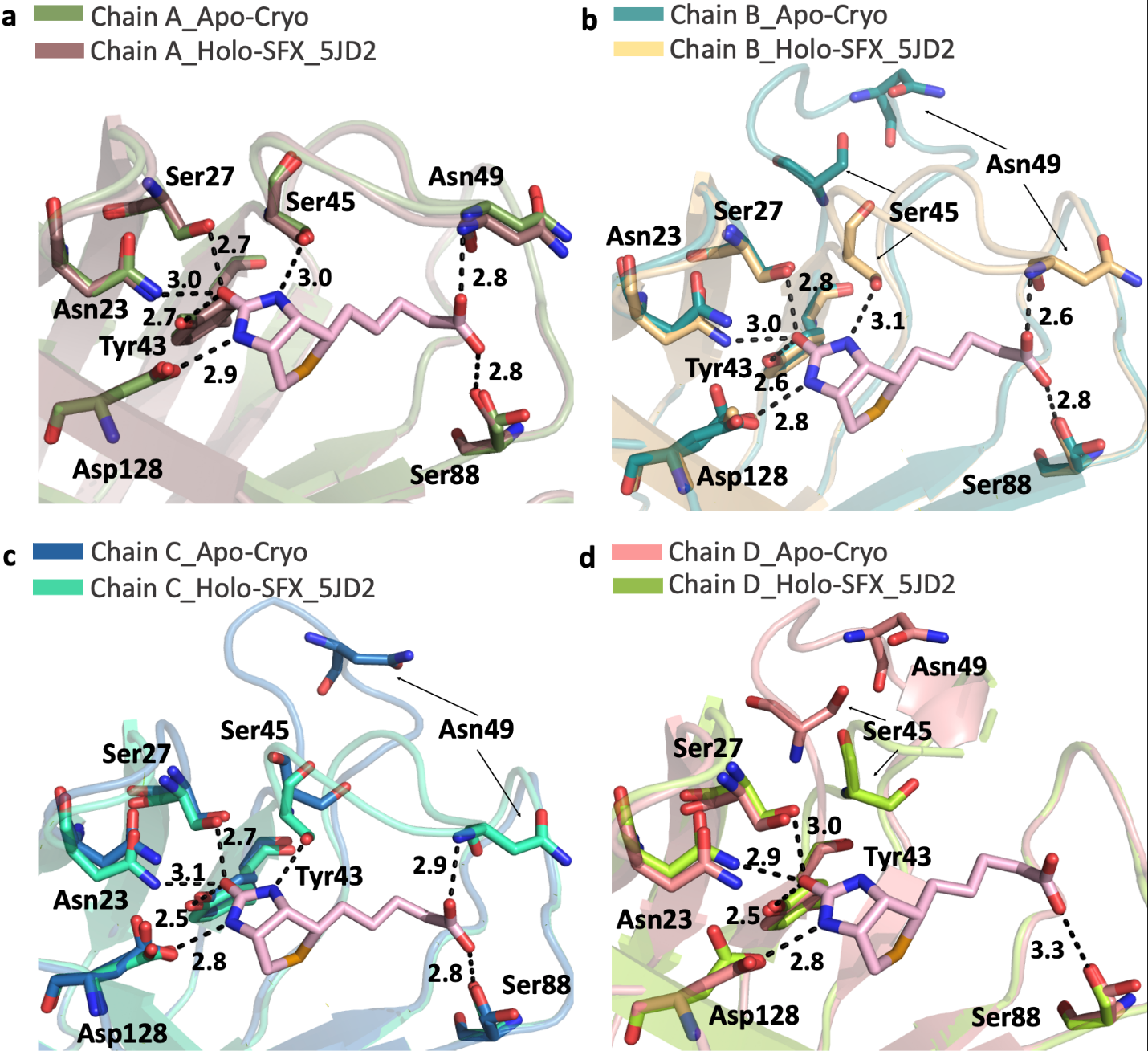


**Supplementary Fig. 11: Superposition of Apo-Cryo structure and Holo-SFX structure (PDB ID: 5JD2) around the binding pocket. (a)** Chain A of both streptavidin structures is superposed with a RMSD of 0. 14 Å. **(b)** Chain B of both streptavidin structures is superposed with a RMSD of 0.14 Å. **(c)** Chain C of both streptavidin structures is superposed with a RMSD of 0.24 Å. **(d)** Chain D of both streptavidin structures is superposed with a RMSD of 0.19 Å. Selenobiotin is colored in light pink and hydrogen bonds are shown with dashed lines.


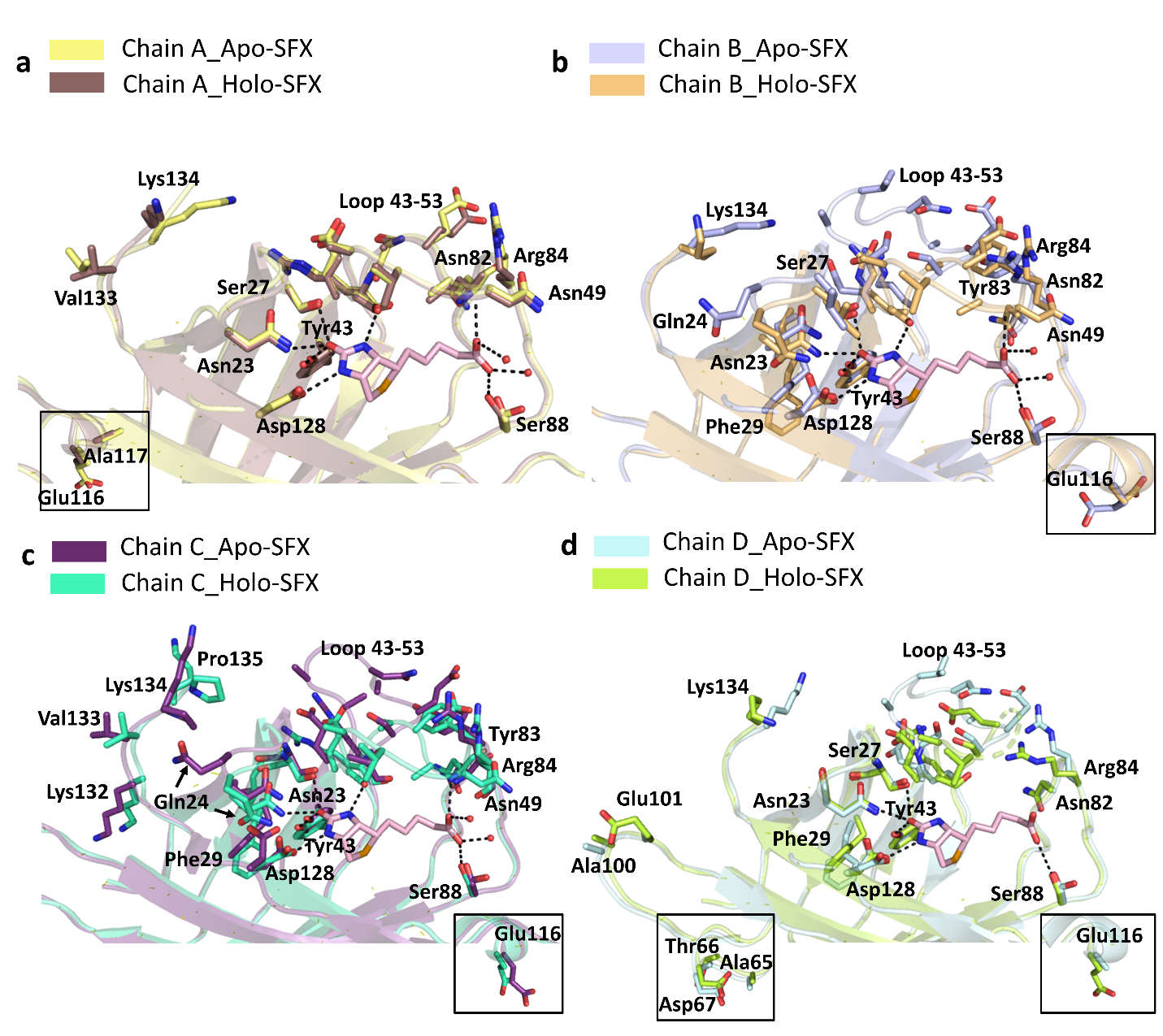


**Supplementary Fig. 12: Side chain conformational differences, which were obtained by overall superposition of Apo-SFX and Holo-SFX (PDB ID:5JD2) structures of streptavidin, display effect of ligand binding** Sidechains with different conformations were shown with sticks and labeled in panel a-d. Residues which are apart from the binding side were represented in the boxes. Each chain was colored according to the previous figure legends. Ligand interactions were shown by black dashed lines.

**
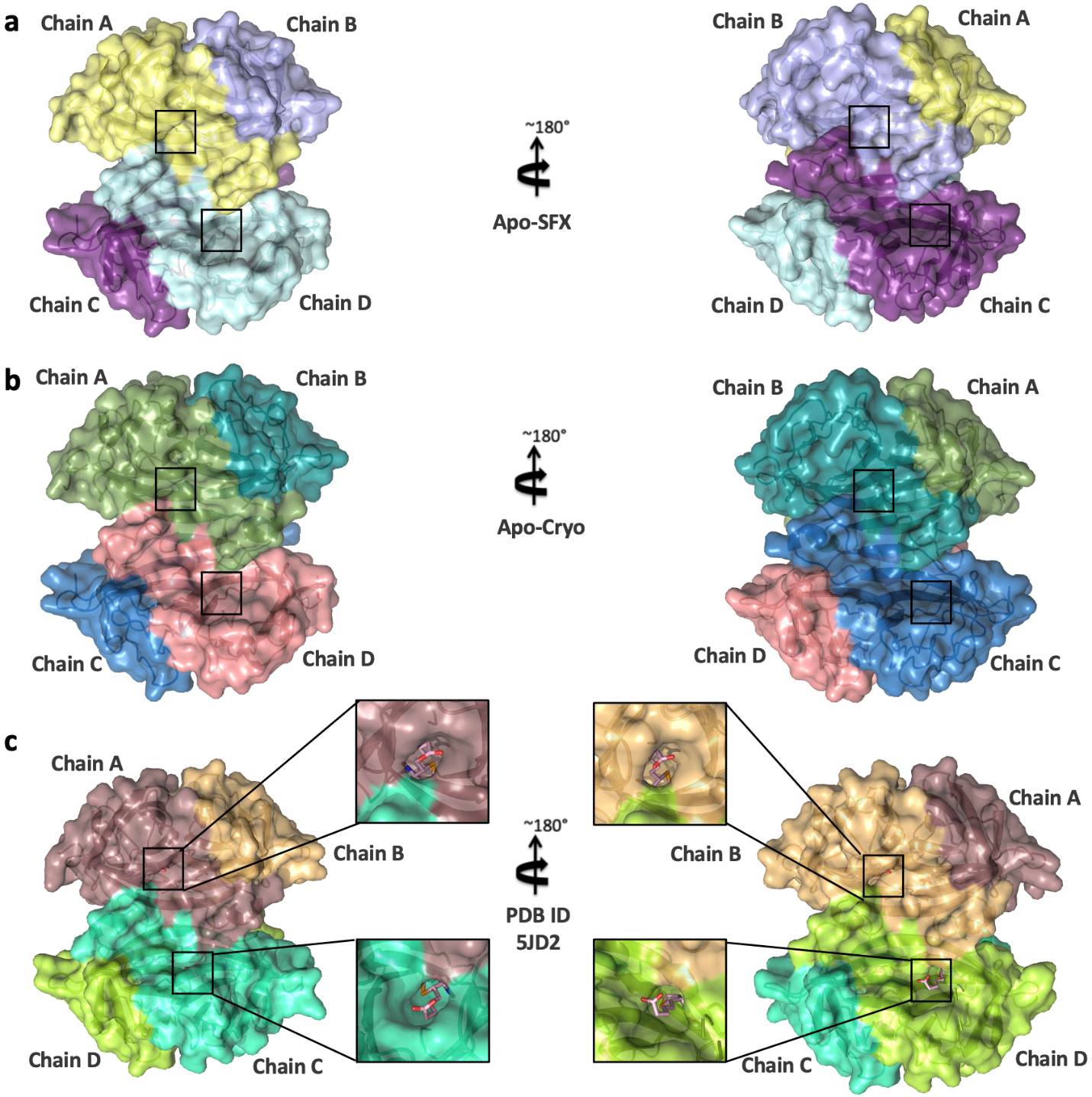
**

**Supplementary Fig. 13: Surface representation of streptavidin structures.** **(a)** Apo-SFX structure of streptavidin **(b)** Apo-Cryo structure of streptavidin **(c)** Holo-SFX structure (PDB ID: 5JD2) are colored based on chain. “The binding pocket for selenobiotin which is colored in light pink is indicated with black squares in the panels.”


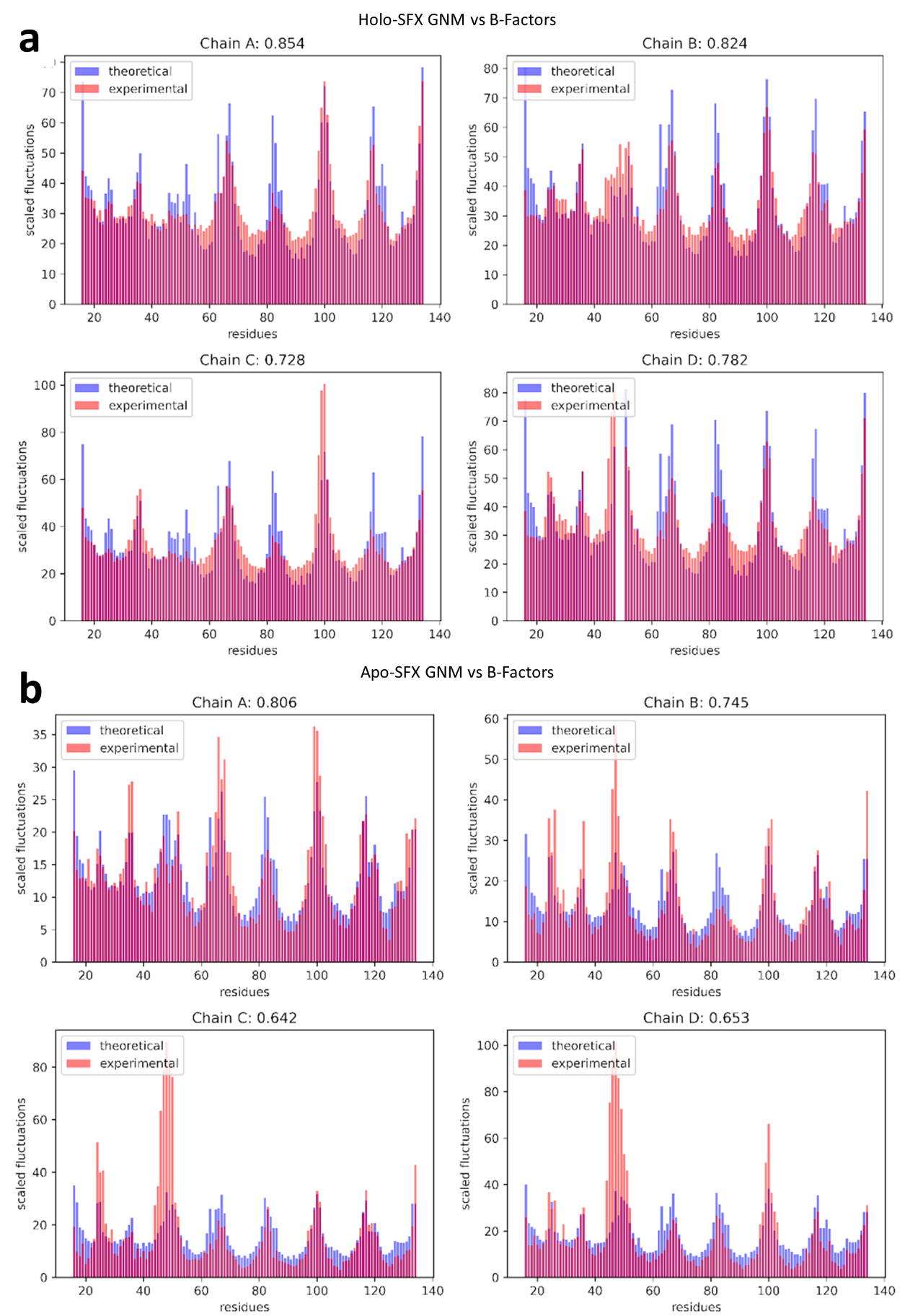


**Supplementary Fig. 14: Theoretical residue fluctuations from GNM compared with the experimental fluctuations**. The correlations between the calculated GNM fluctuations (theoretical) and the B-factors (experimental) are provided for each chain respectively in the results of both Holo-SFX (a) and Apo-SFX (b) structures.


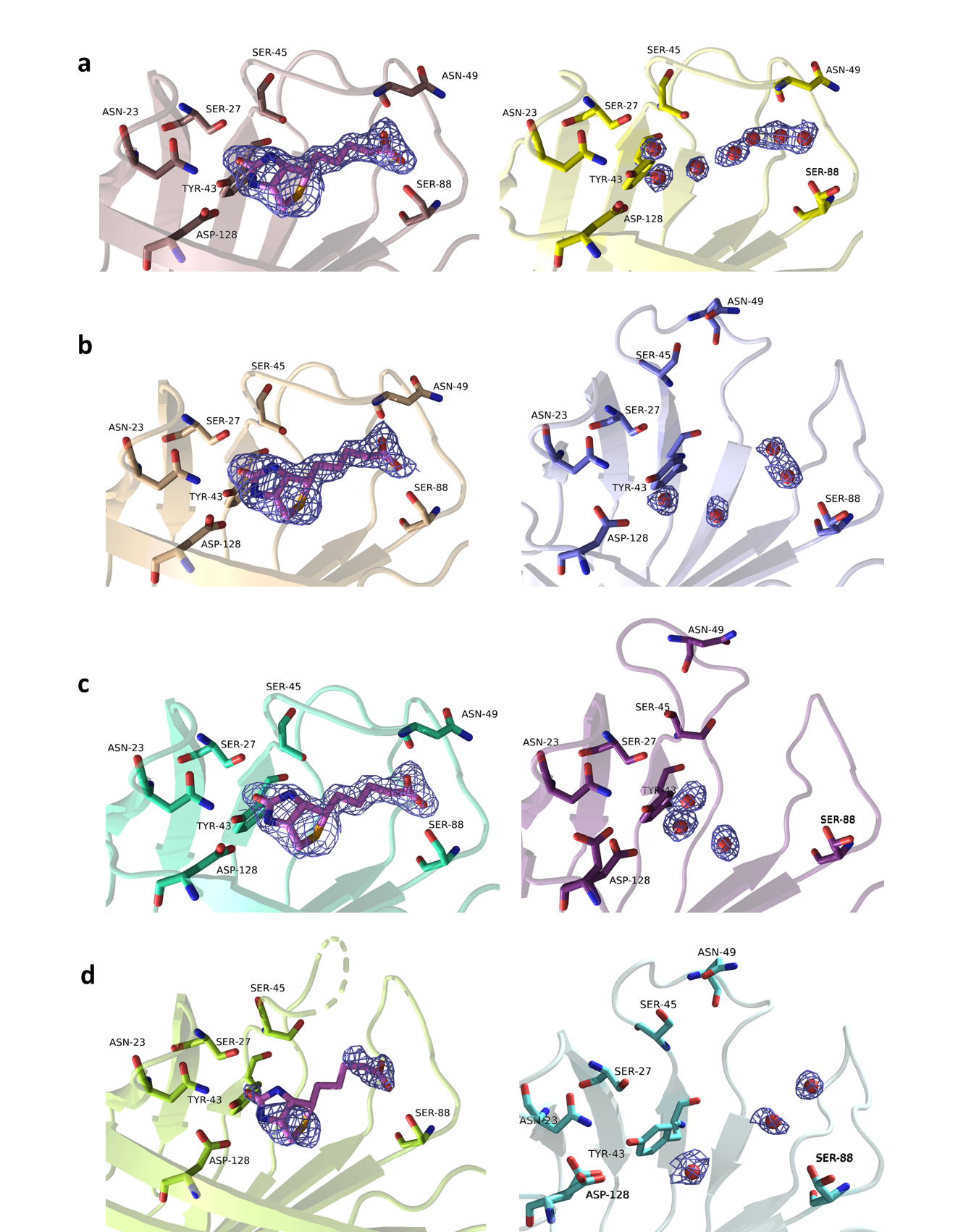


**Supplementary Fig. 15:**  **Electron density maps of all ligands of Holo-SFX (5JD2) and corresponding water molecules at the binding site of Apo-SFX structures.** Electron densities derived from a 2*F*o-*F*c at 1 sigma level and colored in slate. The electron density map of water molecules in the A chain is similar to the selenobiotin ligand electron density map with continuous electron clouds. From the A chain to the D chain (panel a-d), the water-binding activity of the binding pocket of each subunit is asymmetrically different compared to selenobiotin-bound streptavidin corresponding subunits.

**
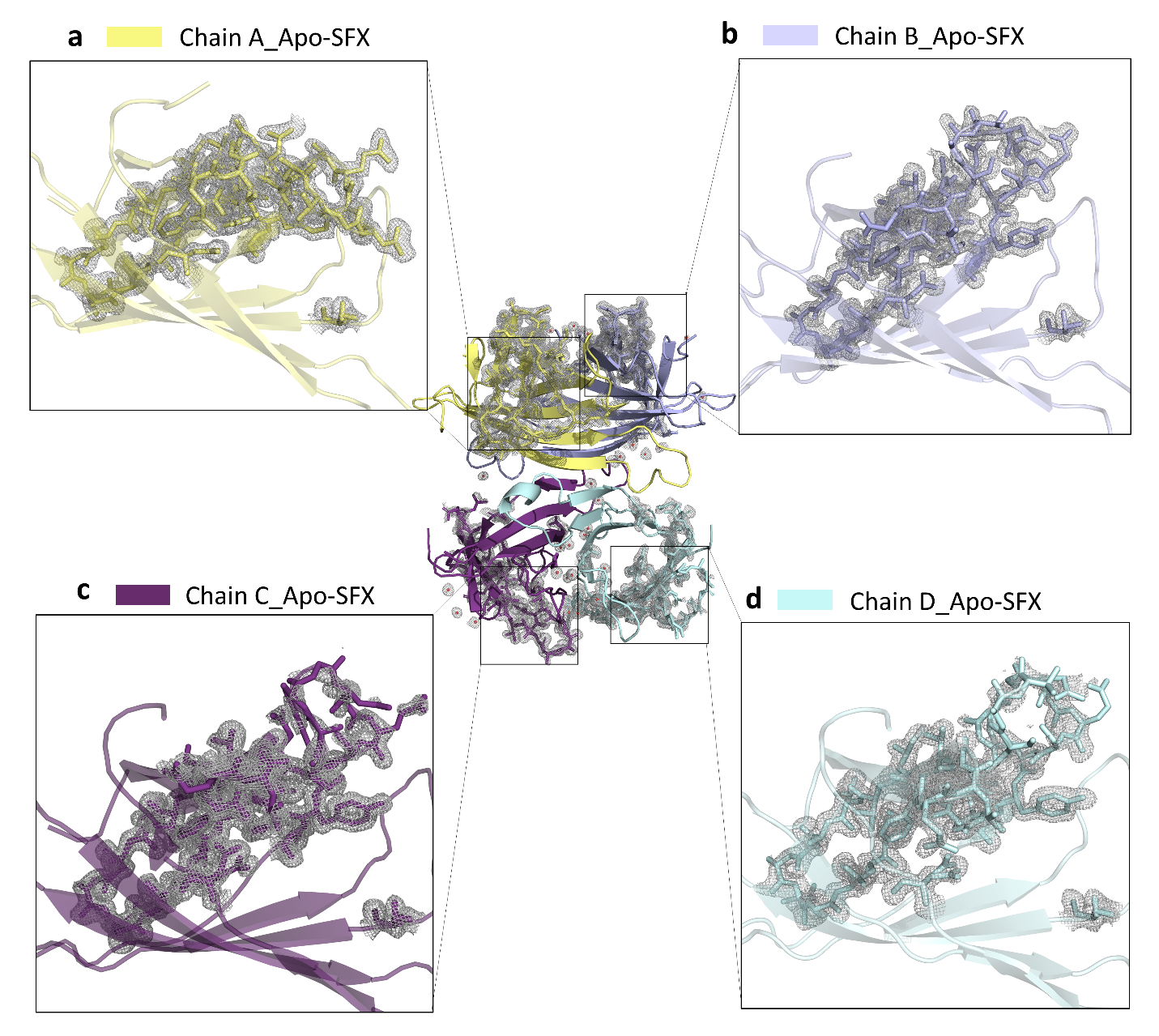
**

**Supplementary Fig. 16: Electron density map of residues which is located near the binding site of the Apo-SFX structure of streptavidin.** 2*F*o-*F*c simulated electron density map is colored in slate in panel a-d. Chain colors are presented as described before. Chain A and B have great overlap with electron density maps. However, some amino acid residues which are on loop with binding site residues are not corresponded with electron density maps in Chain C and Chain D which are disoriented in previous structures.


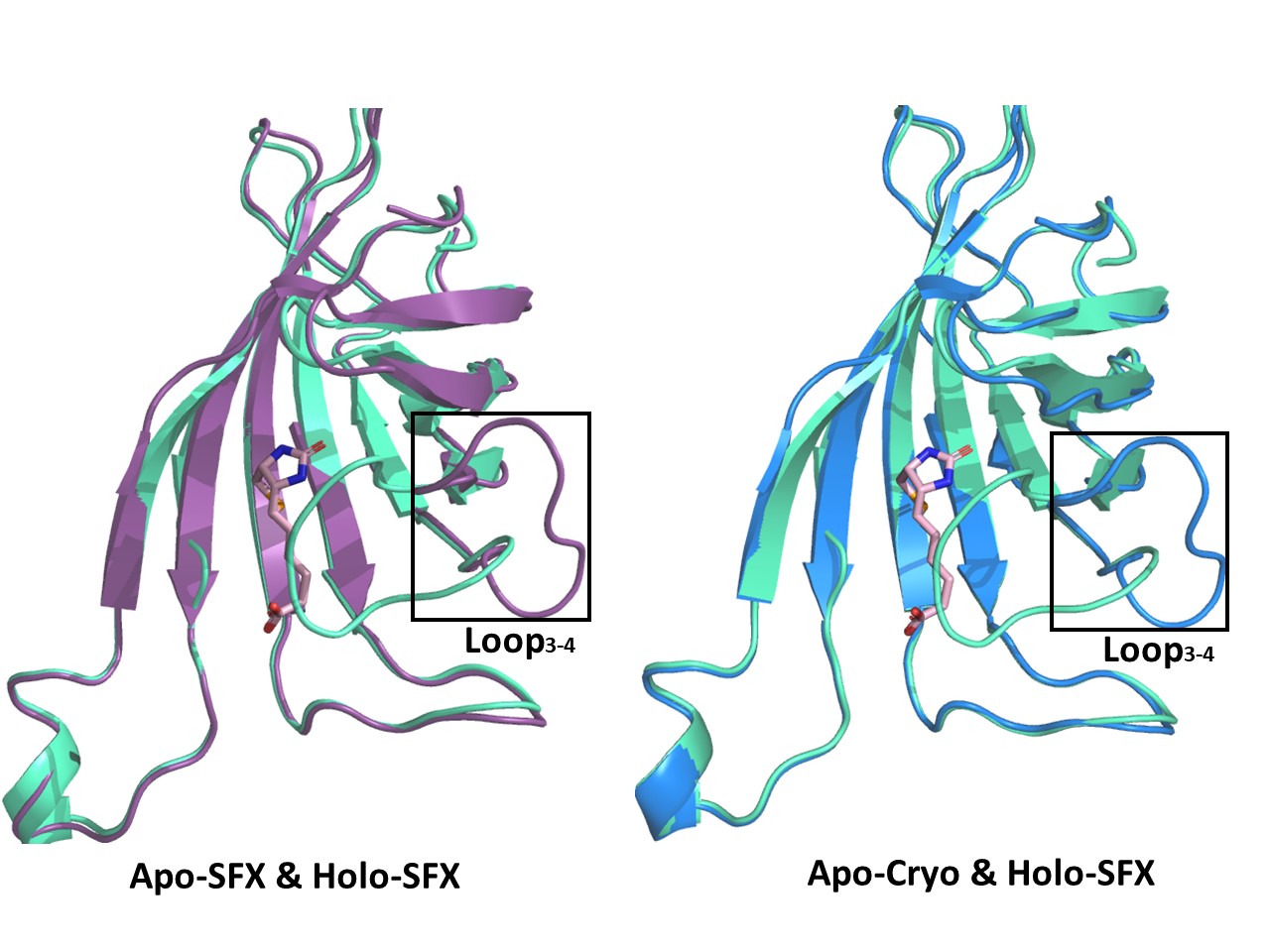


**Supplementary Fig. 17: Representation of loop-closed and loop-open “lid” conformations in superimposed C-chains of the streptavidin**. On the left, loop-open conformations are colored in violet-purple and marine for Apo-SFX in left and Apo-Cryo structures in right, respectively. On the right, loop-closed conformation is colored in green-cyan in Holo-SFX for each C-chains of Apo-SFX structure.

**Supplementary Table 1: Root mean square deviation (RMSD) values between streptavidin structures.** RMSD values for the L3/4 (residues 45-52) are indicated in the parentheses.

| RMSD (Å) of Cα^†^ | | | | |  |
| --- | --- | --- | --- | --- | --- |
|  | Chain A Apo-SFX | Chain B Apo-SFX | Chain C Apo-SFX | Chain D  Apo-SFX |  |
|  |  |  |  |  |  |
| Chain A Apo-Cryo | 0.214 (0.135) | - | - | - |  |
|  |  |  |  |  |  |
| Chain B Apo-Cryo | - | 0.219 (0.119) | - | - |  |
|  |  |  |  |  |  |
| Chain B Apo-Cryo | - | - | 0.228 (0.148) | - |  |
|  |  |  |  |  |  |
| Chain B Apo-Cryo | - | - | - | 0.220 (0.478) |  |
|  |  |  |  |  |  |
| Chain A 6J6K | 0.426 (2.879) | - | - | - |  |
|  |  |  |  |  |  |
| Chain B 6J6K | - | 0.457 (4.484) | - | - |  |
|  |  |  |  |  |  |
| Chain C 6J6K | - | - | 0.481 (3.276) | - |  |
|  |  |  |  |  |  |
| Chain D 6J6K | - | - | - | 0.432 (3.649) |  |
|  |  |  |  |  |  |
| Chain A 6J6J | 0.389 (0.341) | - | - | - |  |
|  |  |  |  |  |  |
| Chain B 6J6J | - | 0.414 (5.076) | - | - |  |
|  |  |  |  |  |  |
| Chain C 6J6J | - | - | 0.411 (2.975) | - |  |
|  |  |  |  |  |  |
| Chain D 6J6J | - | - | - | 0.375 (5.060) |  |
|  |  |  |  |  |  |
| Chain A 5JD2 | 0.269 (0.165) | - | - | - |  |
|  |  |  |  |  |  |
| Chain B 5JD2 | - | 0.213 (4.839) | - | - |  |
|  |  |  |  |  |  |
| Chain C 5JD2 | - | - | 0.360 (2.889) | - |  |
|  |  |  |  |  |  |
| Chain D 5JD2 | - | - | - | 0.262 (2.112) |  |
|  |  |  |  |  |  |

**Supplementary Table 2: Root mean square deviation (RMSD) values between Apo-Cryo and Holo-SFX streptavidin (PDB ID: 5JD2).** RMSD values for the L3/4 (residues 45-52) are indicated in the parentheses.

| RMSD (Å) of Cα^†^ | | | | |  |
| --- | --- | --- | --- | --- | --- |
|  | Chain A Holo-SFX | Chain A Holo-SFX | Chain A Holo-SFX | Chain A Holo-SFX |  |
|  |  |  |  |  |  |
| Chain A Apo-Cryo | 0.144 (0.189) | - | - | - |  |
|  |  |  |  |  |  |
| Chain A Apo-Cryo | - | 0.141 (4.500) | - | - |  |
|  |  |  |  |  |  |
| Chain A Apo-Cryo | - | - | 0.241 (2.876) | - |  |
|  |  |  |  |  |  |
| Chain A Apo-Cryo | - | - | - | 0.190 (2.426) |  |
|  |  |  |  |  |  |

|  |  |  | | |
| --- | --- | --- | --- | --- |
|  |  |  |  |  |
|  |  |  |  |  |
|  |  |  |  |  |
|  |  |  |  |  |
|  |  |  |  |  |

.

70. Laskowski, R. A., Jabłońska, J., Pravda, L., Vařeková, R. S. & Thornton, J. M. PDBsum: structural summaries of PDB entries. *Prot. Sci.* **27**, 129–134 (2018).
