# Supplementary material for: Cooperative allostery and structural dynamics of streptavidin at cryogenic- and ambient-temperature
Source: Commun Biol. 2022 Jan 20;5:73. doi: 10.1038/s42003-021-02903-7 (PMC8776744; doi:10.1038/s42003-021-02903-7)
Supplement: Supplementary file 3 — Reporting Summary [file 42003_2021_2903_MOESM3_ESM.pdf]

nature research

Corresponding author(s): Hasan DeMirci

Last updated by author(s): 10/19/2021

Reporting Summary

Nature Research wishes to improve the reproducibility of the work that we publish. This form provides structure for consistency and transparency in reporting. For further information on Nature Research policies, see our Editorial Policies and the Editorial Policy Checklist.

Statistics

For all statistical analyses, confirm that the following items are present in the figure legend, table legend, main text, or Methods section.

n/a	Confirmed	
☒	☐	The exact sample size (*n*) for each experimental group/condition, given as a discrete number and unit of measurement
☒	☐	A statement on whether measurements were taken from distinct samples or whether the same sample was measured repeatedly
☒	☐	The statistical test(s) used AND whether they are one- or two-sided<br>*Only common tests should be described solely by name; describe more complex techniques in the Methods section.*
☒	☐	A description of all covariates tested
☒	☐	A description of any assumptions or corrections, such as tests of normality and adjustment for multiple comparisons
☒	☐	A full description of the statistical parameters including central tendency (e.g. means) or other basic estimates (e.g. regression coefficient) AND variation (e.g. standard deviation) or associated estimates of uncertainty (e.g. confidence intervals)
☒	☐	For null hypothesis testing, the test statistic (e.g. *F*, *t*, *r*) with confidence intervals, effect sizes, degrees of freedom and *P* value noted<br>*Give P values as exact values whenever suitable.*
☒	☐	For Bayesian analysis, information on the choice of priors and Markov chain Monte Carlo settings
☒	☐	For hierarchical and complex designs, identification of the appropriate level for tests and full reporting of outcomes
☒	☐	Estimates of effect sizes (e.g. Cohen's *d*, Pearson's *r*), indicating how they were calculated

Our web collection on statistics for biologists contains articles on many of the points above.

Software and code

Policy information about availability of computer code

Data collection	SFX data was collected during the LCLS beamtime at SLAC National Accelerator Laboratory through PSOCAKE for determining the initial diffraction geometry of the detector. Synchrotron X-ray diffraction data were collected with a Pilatus 6M detector at the Stanford Synchrotron Radiation Lightsource.
Data analysis	All SFX diffraction patterns were selected using CHEETAH software; indexed by using the CrystFEL software; used as indexing algorithms via XGANDALF, DIRAX, MOSFLM and XDS softwares; integrated and merged using PARTIALATOR; scaled and cut using the TRUNCATE program from the CCP4 suite. All cryo-synchrotron structure, X-ray diffraction data were processed with XDS package for indexing and scaled by using XSCALE.

For manuscripts utilizing custom algorithms or software that are central to the research but not yet described in published literature, software must be made available to editors and reviewers. We strongly encourage code deposition in a community repository (e.g. GitHub). See the Nature Research guidelines for submitting code & software for further information.

Data

Policy information about availability of data

All manuscripts must include a data availability statement. This statement should provide the following information, where applicable:

- Accession codes, unique identifiers, or web links for publicly available datasets
- A list of figures that have associated raw data
- A description of any restrictions on data availability

https://doi.org/10.2210/pdb7EK8/pdb; https://doi.org/10.2210/pdb7EK9/pdb

Field-specific reporting

Please select the one below that is the best fit for your research. If you are not sure, read the appropriate sections before making your selection.

☒ Life sciences ☐ Behavioural & social sciences ☐ Ecological, evolutionary & environmental sciences

For a reference copy of the document with all sections, see nature.com/documents/nr-reporting-summary-flat.pdf

Life sciences study design

All studies must disclose on these points even when the disclosure is negative.

Sample size	Protein samples were purchased without a specific sample size and crystallized by sitting-drop microbatch screening under oil.
Data exclusions	The complete reflection intensity was cut using the TRUNCATE program from the CCP4 suite for SFX structure.
Replication	Replication was provided using 72 well Terasaki crystallization plates.
Randomization	N/A
Blinding	N/A

Reporting for specific materials, systems and methods

We require information from authors about some types of materials, experimental systems and methods used in many studies. Here, indicate whether each material, system or method listed is relevant to your study. If you are not sure if a list item applies to your research, read the appropriate section before selecting a response.

Materials & experimental systems

n/a	Involved in the study
☒	☐ Antibodies
☒	☐ Eukaryotic cell lines
☒	☐ Palaeontology and archaeology
☒	☐ Animals and other organisms
☒	☐ Human research participants
☒	☐ Clinical data
☒	☐ Dual use research of concern

Methods

n/a	Involved in the study
☒	☐ ChIP-seq
☒	☐ Flow cytometry
☒	☐ MRI-based neuroimaging
