# Supplementary material for: Cooperative allostery and structural dynamics of streptavidin at cryogenic- and ambient-temperature
Source: Commun Biol. 2022 Jan 20;5:73. doi: 10.1038/s42003-021-02903-7 (PMC8776744; doi:10.1038/s42003-021-02903-7)
Supplement: Supplementary file 1 — Transparent Peer Review File [file 42003_2021_2903_MOESM1_ESM.pdf]

Peer Review File

Cooperative Allostery and Structural Dynamics of Streptavidin at Cryogenic- and Ambient-temperature

Open Access This file is licensed under a Creative Commons Attribution 4.0 International License, which permits use, sharing, adaptation, distribution and reproduction in any medium or format, as long as you give appropriate credit to the original author(s) and the source, provide a link to the Creative Commons license, and indicate if changes were made. In the cases where the authors are anonymous, such as is the case for the reports of anonymous peer reviewers, author attribution should be to 'Anonymous Referee' followed by a clear attribution to the source work. The images or other third party material in this file are included in the article's Creative Commons license, unless indicated otherwise in a credit line to the material. If material is not included in the article's Creative Commons license and your intended use is not permitted by statutory regulation or exceeds the permitted use, you will need to obtain permission directly from the copyright holder. To view a copy of this license, visit http://creativecommons.org/licenses/by/4.0/.

Reviewers' comments:

Reviewer #1 (Remarks to the Author):

1. This manuscript has some issues. There are multiple typos, omissions and odd expressions. Figures and tables in the supplementary material are not (or rarely) referred to in the main text. Please correct. Can the authors find a way to condense Suppl Tables 2 to 6, there is not so much information in the individual tables.

2. Since all structures are essentially identical, explore, in short, why this is interesting. The Gaussian network decomposition seems to be added to boost significance. This reviewer has doubts that the Gaussian network analysis produces trustworthy results. At least the MSQ fluctuations from GNM can be superposed on the B-factors to see whether that agrees.

3. It appears that this work is the result of an attempt to mix biotin with the SA crystals to follow the binding kinetics, but somehow this did not work (would have been great).

4. What is a "complex SFX structure (PDB ID:5JD2)"? Do the authors mean structure complexed with biotin or selenobiotin?

5. Please define trimmer: "Similarly, Asp61 has correlated motion between residues Thr76 and Thr90 of the neighbor chain on the same trimmer"

6. Please explain "globul": Slowest 10 modes reveals the globul residue motions"

7. Strange sentence: "It must be noted that chain D disorder at 3/4 loop can be clearly seen from the graph with lack of residue data" . What is a graph with lack of residue data?

8. Please rephrase: "Extensive polar interaction network and number of water molecules in direct contact with streptavidin decreased by loosening of L3,4 at binding site (Fig. 7). Presence of a second layer of coordinated water molecules correlated with the loop opening as a "lid" (Fig. 6)."

9. Meaning unclear: "Together with observed conformational changes in the biotin binding pocket (Fig 5), apo streptavidin (PDB ID: 6J6K) obtained by CryoEM was superposed with our SFX structure (Supp Fig 3)."

10. Please explain in more detail: "predisposed cooperative allosterism before binding of the first biotin molecule"

11. Define "vertical": "The next vertical step to better understand"

12. Define "time-stamped", cite available literature. "performing time-stamped structural analysis by using ultrabright and ultrafast XFELs."

13. Please correct expression! As it is written, your crystals diffract with the packing material: "Together with this packing materials and techniques, we were able to get crystals diffracting 1.7 Å resolution."

14. Please explain why molecular replacement with 'phaser' was necessary. What was the reason that model 5JD2 could not be used as a direct initial model that only needed to be adjusted by a rigid body refinement to fit to the XFEL and SSRL data?

15. Please explain and rephrase: "Both MFX and cryo-synchrotron structures were examined to

generate ellipsoid structures based on b-factor with PyMOL." PyMOL does not produce anisotropic B-factors, it displays thermal ellipsoids. Explain how you obtained the anisotropic B-factors?

16. Fig. 4, there different conformations for the Asn49 loop. Describe in the figure caption. Is that functionally significant or a crystal packing artifact?

17. Fig. 5, similar to Fig. 4. Way to short a figure caption. Refer to supplementary table to describe RMSDs.

18. Fig. 6 C, mark the se-biotin.

19. Fig. 7. What is seen here? It is hard to identify the purpose of this figure.

20. Fig. 8. What is an ellipsoid structure? Do the authors mean that thermal ellipsoids were assigned to each (main chain carbon?) atom of the SA structure. What are the red boxes? It is not sufficient to refer to them in the text, they must be explained in the figure caption. Fig. 8C, highlight the biotin in the active site with a different color.

21. Fig. 9. Details such as "atoms N1, C2, C9 and O12 were selected as nodes" do not belong to a figure caption. The cross-correlation heat maps of the SA and SA-biotin seems to be identical. Point out differences, and explain in short in the figure caption. Fig. 9C and Fig. 9D have the same caption "Intrachain correlation differences 5JD2 over Apo_SFX structure (results)". Please check. MSQ fluctuations from the GNM are ok to show, but should be overlaid over observed B-factors (does this agree?).

Reviewer #2 (Remarks to the Author):

The manuscript entitled "Cooperative Allostery and Structural Dynamics of Streptavidin at Cryogenic- and Ambient-temperature" by Ayan et al., presents 2 structures of Apo Streptavidin, one at room temperature (SFX structure) and the second at cryo temperature (synchrotron). The authors performed a Gaussian Network Model analysis on the Apo structure SFX and on a previously solved structure of the Streptavidin bound to selenobiotin.
The GNM analysis provides a dynamic insight of the Streptavidin cooperative allostery, but on the other hand, the GNM study did not seem to need a new apo SFX structure of Streptavidin as plenty of structures of Streptavidin are already available.

Minor comments:
1) Introduction: please add unit for the Kd: "Kd = 10^-13 to 10^-14 M"
2) Introduction: "The conformational dynamics of streptavidin and its interactions shows" I think it should be show and not shows
3) Introduction: "The conformational dynamics of streptavidin and its interactions shows … type of molecule [15]." I don't really understand this sentence, could you please re-formulate it?
4) Materials and methods: Data collection and analysis for cryo-synchrotron studies at SSRL: Is the crystal used for the cryo data from the same pool than the crystals for SFX? Same size?
5) Materials and methods: Data processing for SFX..: Could you please give more details how you define the resolution cut-off for the SFX data with CrystFEL? With CCTBX.XFEL, people use both CC1/2 and redundancy in the high-resolution shell. The C1/2 must decrease in a monotonic fashion and the redundancy for the high-resolution shell must be at least 10-fold.
6) Figure 1: I think you meant: to prevent crystal settling and not to prevent precipitation of proteins
7) Figure 2: please indicate the sigma level of the map
8) Figure 8: space missing "(C)Streptavidin…"
9) Supp Figure 1: in the box next to 1997, I think you meant Trp 120 and. Not Trp 20

10) Supp Figure 1: in the box next to 2020: typo Raidation-damage free instead of Radiation-damage free
11) Supp Figure 1: legend: you probably meant Trp120 and not 20.
12) Supp Figure 1: legend: add space after “2011: Relatively”
13) Supp Figure 2: add sigma level for electronic density map
14) Supp Figure 5: add sigma level for electronic density map
15) Supp Figure 10: authors mentioned 1Fo-Fc map. Is it a typo for 2Fo-Fc map or do you mean Fo-Fc map? Please, add sigma level as well.
16) Supp Figure 11 and 12: are not mentioned at all in the manuscript
17) Supp table 1: please report Rmeas and not Rmerge.
18) Supp table 1: Statistic seems very poor for Cryo-Streptavidin for completeness. I would not publish a structure with 48% completeness in the high-resolution shell neither with 80% completeness overall. I would have a least 90% for the high-resolution shell and >95% overall. The CC1/2 is also quite low. You should cut off the resolution at a lower resolution to have a more acceptable completeness.
19) Supp table 1: Could you add redundancy and completeness for the high-resolution shell for SFX data?
20) Supp table 1: Note 1: One crystal was used for each dataset. This is true for the synchrotron data but untrue for the SFX data.
21) Supp table 2 to 6: I would have like to have the RMSD for the loop 3,4 added to the tables (maybe in parenthesis).
22) To make interaction matrices more readable, I would add where the L3,4 is in the sequence number.

Major comments:

1) From the manuscript, I understand there is no significant difference between the cryo- SSRL structure and the room temperature (RT) MFX structure. How can the authors justify the use of XFEL for solving the RT structure? Radiation damage should not be a main concern as there is no metal/ion in the protein. Using an XFEL for just a RT structure is an overkill as you can do it routinely in synchrotron.
2) What are the new features the SFX structure is bringing compared to the synchrotron structure or what have been done before?
3) The general feeling on this paper is that the GNM study could have been done with previously solved structure. I don’t think the GNM has to use a RT structure. In molecular dynamic simulation, most of the model are issued from cryo- crystallography. For example, 1SWB and 5JD2 are also tetramer and could have been use for this study.

In conclusion, the novelty of this paper does not reside into the structures as they are very similar to previous ones and don’t seem to bring anything new, but in the GNM analysis. The GNM analysis shows the importance of key residues in the mechanism of cooperative allostery. To confirm hypothesis from GNM, the authors suggest at the end of the discussion a time resolved study in the future. This kind of study will definitely justify a serial crystallography approach.

Reviewer #3 (Remarks to the Author):

General Remarks:

Streptavidin is a very important biotechnological tool, being able to capture biotin and biotin derivatives with affinities higher than other commonly used capturing molecules, i.e. antibodies, aptamers, etc. Albeit engineered derivatives were reported to work as monomers or dimers, the wild-type molecule, that coordinates four streptavidin monomers, holds the highest affinity. However, full

occupancy of all four binding sites is still debated. The quaternary structure was solved several times, at varying resolutions, with different ligands, and by different approaches, x-ray crystallography, and later by cryoEM. Streptavidin can be considered a model system to study how multimeric proteins exert their function and how each monomer influences the others is still a focus of study, intrinsically challenging. In streptavidin, Trp120 appears to be important in the intradimer allostery. Ligand binding uses loop 3/4 appears to function as a "lid", closing over the binding pocket when biotin-bound. However, in the apo-state L3/4 appears very flexible and many times unsolved. How the "lid" opening and closing propagates to the binding pocket and how this propagates to the neighboring monomer through trp120 remains under study.

The present study tackles the above questions using the latest systems to resolve crystal structures at ambient temperatures. This new approach is providing novel conformational states at atomic resolution of several proteins and also of highly complex systems as the 30S ribosome. The authors take a further step and solve the same apo structure using classical x-ray diffraction at cryogenic conditions. In addition, the authors compare their new structures to previously reported structures of streptavidin complexed with seleno-biotin/biotin or other apo states. Ayan et al. describe defined electron densities for L3/4 for the Apo structures, at both, ambient and cryogenic conditions. Interestingly, not all L3/4 loops appeared in the open state. 3 out of 4 lids appeared open while one was similar to biotin-bound closed lid. A comparison with the seleno-biotin bound structure, also obtained at ambient temperature, indicates a certain asymmetry among monomers. In the bound state, 3 out 4 lids were closed while one stayed open. Another interesting finding relies on the intramonomer conformational coupling between the lid and the binding site. For instance, the density of positive charges in the binding pocket decreases if the lid is closed, similarly to when biotin is bound. This indicates that redistribution of charges in the binding pocket is not mediated by biotin. Also, coordinated water molecules are affected, yet more dependent on biotin binding rather than the lid state. In general, the coordinated number of water molecules appears reduced when biotin bound. The Gaussian Network Model analysis highlights several intra- and interchain correlated conformational changes.

Altogether, the work of the DeMirci group provides new results and analysis defining how the tetrameric streptavidin orchestrates to capture biotin. The work is technically well executed by an expert team. Nevertheless, the manuscript requires a number of adjustments, here you can find some suggestions:

Major concerns:
Manuscript:
1) The main goal and objectives of the study are not clearly introduced to the reader. Also, the reason behind the necessity to solve the structure at ambient or physiological temperatures is not clear, what is expected? How is this approach providing new insights into other complexes/proteins? Why is it important to solve the Apo state? Introducing the reader to the above can tremendously increase the clarity of the manuscript.
2) The results section appears very slim. Seven main figures and seven supplementary figures are packed into two pages of text. This section could be extended with better descriptions of the main findings. Now they appear hidden within other less important results or comments. For example: in the 2nd paragraph, "We observed additional electron density that belongs to the residues in the L3,4 region which were not modeled in previous studies". This deserves a more detailed description of the result. Here go some examples where the authors could expand and explain better:
3) Page 5: "… we observed the minor conformational changes ..." The authors do not define or mention how many Ås imply minor of major conformational changes.
4) Page 7: "Slowest 10 modes reveals the globul residue motions", I am not sure if the authors meant "global" or "globular".
5) Page 8: "These results confirm biotin binding may not have a stabilization effect however contribute to the allostery of streptavidin" This is not clear,the authors may need to rephrase this sentence. Allostery is a property of certain proteins, then, it is not clear how the biotin binding might contribute

to the allostery of streptavidin. Ligands can cause allosteric responses, they do not contribute to allostery.
6) Page 9: "However, our atomic resolution structural and GNM data suggest that there is a predisposed cooperative allosterism before binding of the first biotin molecule."
This sentence is not clear, do authors mean that "predisposed cooperative allosterism" is a property of the streptavidin? Or do they imply the protein has a certain predisposition to bind, particularly, biotin with cooperative allosterism?
7) In general, the results segment could be described in more detail, likely considering rearranging some of the main figures. A good rule of thumb is that each figure should relate to a results segment, supporting a well described finding.

Figures:
8) Figures 1 does not add to the manuscript since the novel approach is not the main focus of the paper. Perhaps, Figure 1 could go to supplementary figures. An alternative would be to combine figure 1 with a scheme of the study indicating the main question to be addressed.
9) Figure 4: comparison of the SFX structure with the apo structure obtained by cryoEM, is it necessary in the main figures? It is not used for any other further comparison (i.e. Fig. 6, Fig. 8, Fig. 9). Perhaps it could be moved to supplementary figures.
10) Supp Fig 9 adds interesting information which together with Fig 6, and Fig 8 could become a main figure summarizing how the binding pocket topography, charges, elasticity and water coordination changes as a function of, for example, lid state, or biotin-bound to unbound.

Scientific:
11) I remained puzzled about the similarities between the ambient and cryo structures. What defines the conformational state of streptavidin? How much the crystallization process is influencing? What is the contribution of the increased temperature?
12) How do the new apo structures compare to other Apo-states that had been resolved previously? The comparison with PDB: 6J6K, obtained by cryoEM, shows different conformations of L3/4, all closed albeit obtained the Apo state (Fig. 4), how do you interpret this?

13) Page 9: "Together with observed conformational changes in the biotin binding pocket (Fig 5), apo streptavidin (PDB ID: 6J6K) obtained by CryoEM was superposed with our SFX structure (Supp Fig 3). The comparison of the structures suggests that the two techniques can capture alternative binding conformations and expand the conformational space sampling of the active site loop."

Taking into account that the RMSD between the binding site residues of apo and holo structures is less than 0.4A, how can the authors assure these differences are only usual fluctuations of the residues, or error during the experimental elucidation of the structures, or they are actually real differences between the apo and holo states of streptavidin?

14) During the GNM analysis, the authors mention they used the 10 slowest modes to be correlated with the global motions of the protein, and the 10 fastest modes for localized motions. So, how many modes were in total calculated by the GNM analysis?

Minor points:
15) Supplementary Figure 1 refers to structures obtained in "2020", intended to the structures of this work. Please update to 2021 and indicate that they belong to "this work". Also, "2014" in the captions should be bold to maintain consistency.
16) The units of the KD is missing in 4th line of the second paragraph of the introduction. Should read: KD = 10-14 to 10-15 M
17) Reference 10 suggests that modifications in biotin may cause a "more "disordered" L 3/4 loop in avidin, rather than in streptavidin. Although both are highly conserved, there are differences, specially in L 3/4. Please, indicate what is the important role of L3/4 mentioned in the last sentence of the second paragraph of the introduction.

18) Nomenclature of the different structures analyzed: please keep consistency, specially related to the SFX (ambient) structures. Sometimes it is called "ambient structure", "SFX structure" "APO-SFX", and so on.
19) Bound not bounded, correct along the text.

Point by Point Responses to Reviewers' comments

Please find below our point-by-point responses to our Reviewers' comments written in blue text. All changes on the manuscript are also highlighted with yellow color on the main text and supplementary files.

Reviewer #1 (Remarks to the Author):

This manuscript has some issues. There are multiple typos, omissions and odd expressions. Figures and tables in the supplementary material are not (or rarely) referred to in the main text. Please correct.

RESPONSE: We sincerely apologize for all the typos and other issues. We extensively reviewed our manuscript and fixed all the existing points raised by our Reviewer 1.

1. Can the authors find a way to condense Supp Tables 2 to 6, there is not so much information in the individual tables.

RESPONSE: As suggested by our Reviewer, we merged these Tables.

2. Since all structures are essentially identical, explore, in short, why this is interesting. The Gaussian network decomposition seems to be added to boost significance. This reviewer has doubts that the Gaussian network analysis produces trustworthy results. At least the MSQ fluctuations from GNM can be superposed on the B-factors to see whether that agrees.

RESPONSE: The structures are similar however they are not identical because the binding loop of selenobiotin (3/4 loop) conformations is distinct in the Apo_SFX vs 5JD2 (holo-SFX) structure. These differences in loop motions can also be detected with GNM analysis by analyzing the protein's global and local motions. Due to differences in the conformations, the network models are also distinct from each other and the Normal Mode Analysis of these models supports these data. Particularly, the binding of selenobiotin affects the residue fluctuations and their correlations which we discussed in detail. We actually selected the cutoff distance for calculating GNM based on the correlation with the b-factors in 5JD2. We rewrote the method section to clarify the steps in the calculation process and provided the missing details. We compared the theoretical temperature factors calculated with GNM to the experimental b-factors also by checking the Pearson correlation between them: the overall correlation was 0.785 in the 5JD2 structure and 0.646 in Apo-SFX. Higher cutoff in Apo-SFX would probably increase correlation at the binding site residues, however, we wanted to maintain compatibility in the analysis with the same parameter selection. As

the reviewer kindly requested, we show their superimposition in the figures below with the correlations for each chain calculated separately:

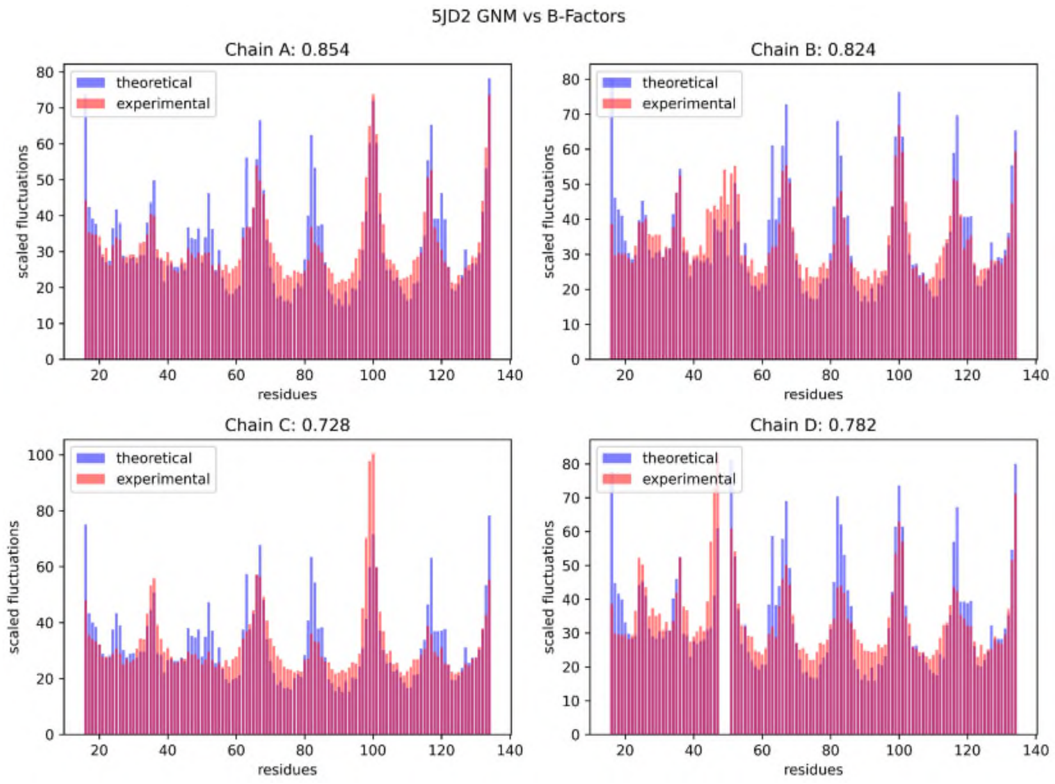

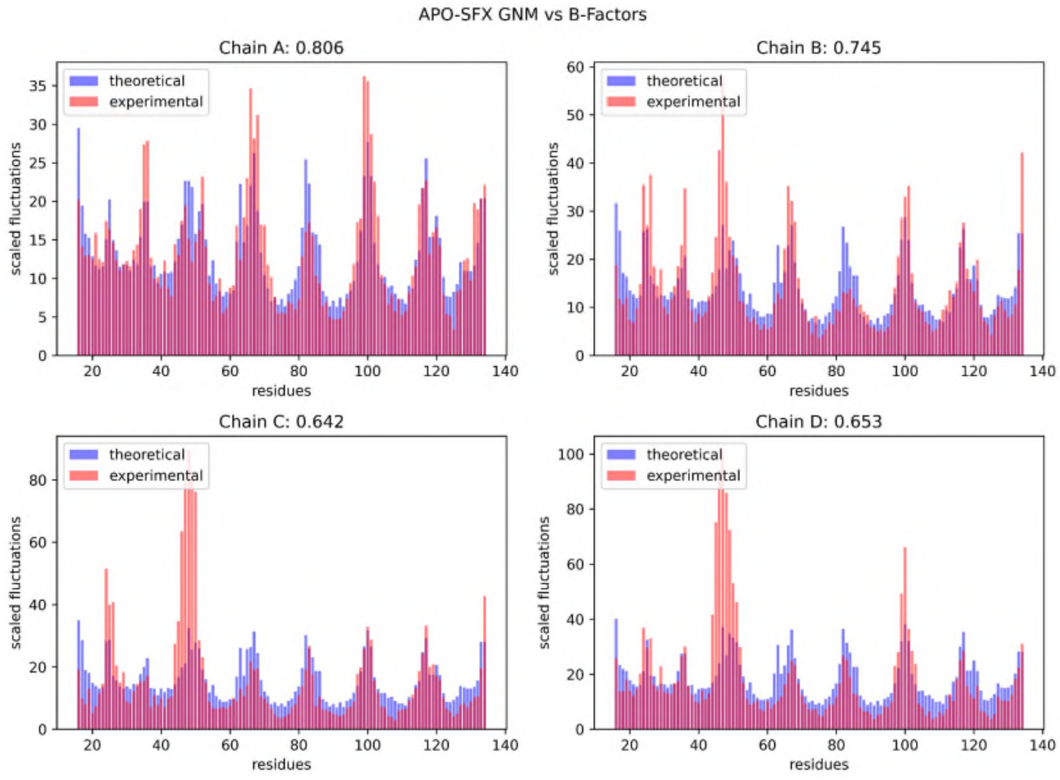

3. It appears that this work is the result of an attempt to mix biotin with the SA crystals to follow the binding kinetics, but somehow this did not work (would have been great).

RESPONSE: Thank you for reviewer 1's valuable comment and we apologize for not being able to explain our story line clearly. Our next obvious step as indicated by our Reviewer is to better understand the details of the binding kinetics and cooperative allosterism by performing time-resolved structural analysis by using the streptavidin-biotin fast-mixing kineto-crystallography technique.

We would like to explain the main purpose of the current study more explicitly as follows:

Streptavidin is a paradigm protein complex system with the highest affinity for its ligand among other proteins in its class. This reputation in affinity is due to the coordination of all monomers of streptavidin compared to the function of its subunits alone. Therefore, the binding kinetics of the streptavidin to the substrate is closely related to the occupancy of all four binding sites. Although a wide variety of structural studies have been carried out, the mystery of this high affinity still remains unsolved. In this study, we not only elucidated the high-resolution apo-structure of the protein at cryogenic and near-physiological temperatures but also emphasized several intra- and interchain correlated motions with GNM analysis. The dynamics of the structure obtained by the GNM analysis and the extensive polar interaction network in the binding region obtained by the Apo-SFX structure confirm for the first time presence of a novel cooperative allosterism of streptavidin. The choice of the Apo-structure in the study is, naturally, to observe the non-ligand-bound L3,4 conformation of this model system and compare it with our previous holo-SFX structure.

In the X-ray crystallographic aspect of our study, the intermonomer function of Trp120 on structural cooperation and the effect of this on the loop 3/4 conformation, which acts like a "lid", was emphasized. Interestingly, not all L3/4 loops in the apo-structure were open. Only 3 lids were open and one was in a closed conformation such as ligand-bound. We observed that this is related to the number of water molecules and hydrogen bonds in the binding site. When the lid is closed, we observe a decreased density of positive charges in the binding pocket, just as in the presence of the ligand. This demonstrates that redistribution of charges, the interaction of the coordinated water molecules and extensive polar interactions network in the binding pocket is not regulated by biotin but regulated with cooperative allosterism property of streptavidin. To confirm this hypothesis, we performed extensive GNM analysis, using both our apo-structures and our previous holo-structure. The asymmetry between monomers in the ligand-bound structure and several intra- & intermonomer-related correlated motions with allostery provided more robust structural dynamics that define how streptavidin regulates to interaction with biotin via GNM analysis.

All these results and our literature comparisons demonstrate the novel cooperative allosterism of streptavidin to bind to its ligand through water molecules that mimic the substrate and provide the polar network.

4. What is a “complex SFX structure (PDB ID:5JD2)”? Do the authors mean structure complexed with biotin or selenobiotin?

RESPONSE: We want to thank reviewer 1 for highlighting this mistake. This part has been changed to "The superposition of our Apo-SFX structure was performed by using the cryoEM structure complexed with biotin (PDB ID: 6J6J) (Supplementary Fig. 7 & Supplementary Table 2) and SFX structure complexed with selenobiotin (PDB ID:5JD2) (Fig 3, Supplementary Table 2)".

5. Please define trimmer: “Similarly, Asp61 has correlated motion between residues Thr76 and Thr90 of the neighbor chain on the same trimmer”

RESPONSE: As our reviewer 1 pointed out, now this part has been changed to “Similarly, Asp61 has correlated motion between residues Thr76 and Thr90 of the neighbor chain on the same trimer (Fig 8A, B))

6. Please explain “globul”: Slowest 10 modes reveals the globul residue motions”

RESPONSE: We want to thank reviewer 1 for highlighting this mistake. This part has been changed to “The weighted 10 slowest mode results which reveal the protein’s global motions are provided in Fig. 8E”

7. Strange sentence: “It must be noted that chain D disorder at 3/4 loop can be clearly seen from the graph with lack of residue data (Fig. 8E)”. What is a graph with lack of residue data?

RESPONSE: We want to thank reviewer 1 for highlighting this issue. This part has been changed to “It must be noted that chain D disorder at 3/4 loop can be clearly seen from the graph (Fig. 8E)”

8. Please rephrase: “Extensive polar interaction network and number of water molecules in direct contact with streptavidin decreased by loosening of L3,4 at binding site (Fig. 7). Presence of a second layer of coordinated water molecules correlated with the loop opening as a “lid” (Fig. 6).”

RESPONSE: We would like to thank reviewer 1 for pointing out this obscure part. Text has been changed to “The polar interaction networks and number of water molecules in contact with streptavidin are directly related to the L3,4 conformation. It has a closed conformation through increased polar interactions at the binding site (Fig. 5 & Supplementary Fig. 12A). However, decreases in coordinated water molecules are

associated with an open conformation of the loop, acting as a "lid" (Fig. 3 & Supplementary Fig. 12 B,C,D).”

9. Meaning unclear: “Together with observed conformational changes in the biotin binding pocket (Fig 5), apo streptavidin (PDB ID: 6J6K) obtained by CryoEM was superposed with our SFX structure (Supp Fig 3).”

RESPONSE: We would like to thank reviewer 1 for highlighting this obscure phrase. This sentence has been changed to “Similar to the selenobiotin-bound structure (Fig. 3), the cryo-EM structure of streptavidin in complex with biotin (PDB ID: 6j6j) were superposed with our Apo-SFX structure (Supplementary Fig. 6).”

10. Please explain in more detail: “predisposed cooperative allosterism before binding of the first biotin molecule”

RESPONSE: We apologize for this vague sentence. We fixed it as “However, our atomic-resolution structural and GNM data suggest that there is a predisposed property of streptavidin cooperative allosterism before binding of the first biotin molecule.”

11. Define “vertical”: “The next vertical step to better understand”

RESPONSE: This part has been changed to “The next step to better understand…”

12. Define “time-stamped”, cite available literature. “Performing time-stamped structural analysis by using ultrabright and ultrafast XFELs.”

RESPONSE: This part has been changed to “...performing time-resolved structural analysis by using ultrabright and ultrafast XFELs [44]” We included Stagno et al’s milestone 2016 time-resolved riboswitch paper as the reference.

13. Please correct expression! As it is written, your crystals diffract with the packing material: “Together with this packing materials and techniques, we were able to get crystals diffracting 1.7 Å resolution.”

RESPONSE: ‘’Transport packing by using large quantities of cottons prevented physical damage of the crystals and successfully transportation to XFEL, was followed by diffraction to 1.7 Å resolution.’’

14. Please explain why molecular replacement with ‘phaser’ was necessary. What was the reason that model 5JD2 could not be used as a direct initial model that only needed to be adjusted by a rigid body refinement to fit to the XFEL and SSRL data?

RESPONSE: There is significant unit cell dimension and angle changes which prevents direct rigid-body refinement and necessitated molecular replacement. Please see below corresponding unit cell dimensions and angles of the Asymmetric Unit Cell.

Unit cell dimensions and angles of 5JD2: 50.84, 98.52, 53.43 and 90, 112.38, 90
Unit cell dimensions of Apo_SFX: 47.40, 87.70, 58.90 and 90.00, 98.90, 90.00
Unit cell dimensions of cryo-synchrotron structure: 46.36, 85.76, 58.13 and 90.00, 98.71, 90.00

15. Please explain and rephrase: “Both MFX and cryo-synchrotron structures were examined to generate ellipsoid structures based on b-factor with PyMOL.” PyMOL does not produce anisotropic B-factors, it displays thermal ellipsoids. Explain how you obtained the anisotropic B-factors?

RESPONSE: We would like to thank reviewer 1 for his/her comment. We used anisotropic B-factors generated by PHENIX for obtaining ellipsoid structure. Like the reviewer mentioned, we only visualized the thermal ellipsoids of our structures and colored them in PyMOL and displayed the stable and flexible regions for both SFX and cryo-synchrotron structures. A detailed explanation is added to the legend of Figure 6 to mark the ‘’thermal’’ ellipsoid.

16. Fig. 4, there are different conformations for the Asn49 loop. Describe in the figure caption. Is that functionally significant or a crystal packing artifact?

RESPONSE: We would like to thank Reviewer 1 for his/her comment. Due to another suggestion, we moved this figure to supplementary figures as Supplementary Fig. 6. Different loop conformation is observed for both SFX and cryo structures compared to the cryo-EM structure (PDB ID:6J6K) (Supplementary Fig. 6). Based on our structures, the electron density of the loop is well defined to describe the position of the loop (Supplementary Fig. 13). It is the reason that we emphasize this issue as functionally significant in this paper. It can provide invaluable information for future studies. Asn 49 loop is called loop 3/4 which is located near the binding site may act as a ‘’lid’’ to regulate the function of streptavidin in the presence and absence of ligand.

17. Fig. 5, similar to Fig. 4. Way to short a figure caption. Refer to supplementary table to describe RMSDs.

RESPONSE: Due to the renumbering of the figures, the former figure 5 is now figure 3 in the new version of the manuscript. We extended the legend of the figure with an explanation of the outcomes of "The 3/4 loop open[ing] as a "lid" without selenobiotin binding. Binding of selenobiotin is not symmetric for all four monomers which represent cooperativity." Moreover, "Hydrogen bonds are shown with black dashed lines and their corresponding distance as a unit of Angstrom (Å)." was added for a better understanding.

18. Fig. 6 C, mark the se-biotin.

RESPONSE: Due to the renumbering of the figures, former figure 6C is now figure 4C in the new version of the manuscript. As suggested, we marked seleno-biotin in the relevant figure 4C.

19. Fig. 7. What is seen here? It is hard to identify the purpose of this figure.

RESPONSE: Due to the renumbering of the figures, Fig. 7 is figure 5 in the new version of the manuscript. We extend the legend of the figure with "Coordinated water molecules within the binding pocket were altered and polar interactions were reduced with loop opening in APO_SFX structure. All polar interactions were observed within 3.6 Å." and "Water molecules were indicated with red-colored spheres." for clarification. Moreover, for a better image, the transparency of the cartoon is increased, and water molecules were shown with bigger spheres.

20. Fig. 8. What is an ellipsoid structure? Do the authors mean that thermal ellipsoids were assigned to each (main chain carbon?) atom of the SA structure. What are the red boxes? It is not sufficient to refer to them in the text, they must be explained in the figure caption. Fig. 8C, highlight the biotin in the active site with a different color.

RESPONSE: Due to renumbering of the figures, former figure 8 is now figure 6 in the new version of manuscript. The thermal ellipsoid for both SFX and cryo structure is presented to reveal the stable and flexible regions of the protein colored based on b-factors. The missing details are mentioned in the legend of figure 6 (new numbering order). Red boxes indicate the flexible (red/orange color) and stable (blue/green color) regions on the structure. Based on our reviewer's comment, the biotin molecule is highlighted with different colors to emphasize the active site.

21. Fig. 9. Details such as “atoms N1, C2, C9 and O12 were selected as nodes” do not belong to a figure caption. The cross-correlation heat maps of the SA and SA-biotin seems to be identical. Point out differences, and explain in short in the figure caption. Fig. 9C and Fig. 9D have the same caption “Intrachain correlation differences 5JD2 over Apo_SFX structure (results)”. Please check. MSQ fluctuations from the GNM are ok to show, but should be overlaid over observed B-factors (does this agree?).

RESPONSE: Due to renumbering of the figures, former figure 9 is now figure 8 in the new version of manuscript. We deleted the “atoms N1, C2, C9 and O12 were selected as nodes” section. However, we would like to mention that 5JD2 and the SFX structure of streptavidin do not have the same cross-correlation maps. The differences between the two maps were already indicated at Fig 8C and 8D accordingly (inter- or intra-molecular interactions of the selected chains). Moreover, we need to clarify, with all due respect, that Fig 8D and 8C do not have the same caption. We further modified it to emphasize this as follows “C) Differences between the intrachain cross-correlations of 5JD2 chain B over Apo_SFX structure chain A. D) Differences in the interchain cross-correlations of 5JD2 over Apo_SFX structure at cross-sections of chains A and B.” As we mentioned in Comment 2, the theoretical fluctuations calculated with GNM agreed with the experimental b-factors: 0.785 correlation in the 5JD2 structure and 0.646 in Apo-SFX. We provided the figure with b-factors overlaid as Supplementary Fig. 12.

****

Reviewer #2 (Remarks to the Author):

The manuscript entitled “Cooperative Allostery and Structural Dynamics of Streptavidin at Cryogenic- and Ambient-temperature” by Ayan et al., presents 2 structures of Apo Streptavidin, one at room temperature (SFX structure) and the second at cryo temperature (synchrotron). The authors performed a Gaussian Network Model analysis on the Apo structure SFX and on a previously solved structure of the Streptavidin bound to selenobiotin.The GNM analysis provides a dynamic insight of the Streptavidin cooperative allostery, but on the other hand, the GNM study did not seem to need a new apo SFX structure of Streptavidin as plenty of structures of Streptavidin are already available.

Minor comments:

1) Introduction: please add unit for the Kd: “Kd = 10^-13 to 10^-14 M”

RESPONSE: We would like to thank reviewer 2 for highlighting this missing part. Unit for Kd is added in the main text.

2) Introduction: "The conformational dynamics of streptavidin and its interactions shows" I think it should be show and not shows

RESPONSE: The grammar mistake which is indicated in minor comment 2 is corrected in the main text.

3) Introduction: "The conformational dynamics of streptavidin and its interactions shows … type of molecule [15]." I don't really understand this sentence, could you please re-formulate it?

RESPONSE: The sentence re-formulated as " The structural dynamics of streptavidin and its interaction with small molecules demonstrate that we have a greater understanding of its structure-function relationship, which makes it easy to "plug and play" type of molecule.

4) Materials and methods: Data collection and analysis for cryo-synchrotron studies at SSRL: Is the crystal used for the cryo data from the same pool than the crystals for SFX? Same size?

RESPONSE: The answer of question 4 from reviewer 2 is no. The crystals that are used for cryo and SFX structure come from the same batch, however, obtained with different crystallization techniques. While the large cryo-synchrotron crystal obtained with microbatch under oil, the micro crystals for SFX obtained by batch method. Additionally, unit cell parameters for both cryo and apo structure were indicated in Supplementary Table 1.

5) Materials and methods: Data processing for SFX..: Could you please give more details how you define the resolution cut-off for the SFX data with CrystFEL? With CCTBX.XFEL, people use both CC1/2 and redundancy in the high-resolution shell. The C1/2 must decrease in a monotonic fashion and the redundancy for the high-resolution shell must be at least 10-fold.

RESPONSE: Resolution cut-off for all crystallography data is generally based on CC* and not CC1/2. CC* should be > 0.5 (it is 0.87). The 10-fold redundancy criteria may be something the cctbx users abide by, but it was not considered in this paper due to data processing with CrystFEL for our SFX data. The redundancy in the high-resolution shell

for SFX data is 63. So, if anything our SFX data is of higher resolution, the resolution cut-off is based on the Wilson plot (which is independent from CrystFEL processing) rather than moving the detector forward (it was not possible because of the chamber set-up). The Wilson statistics confirmed a good fit to the data down to a resolution of 1.7 Å.

6) Figure 1: I think you meant: to prevent crystal settling and not to prevent precipitation of proteins

RESPONSE: We would like to thank reviewer 2 for his/her comment. Figure 1 is replaced as Supplementary Fig. 3. The legend of Supplementary Fig. 3 is corrected as ‘’crystal settling’’.

7) Figure 2: please indicate the sigma level of the map

RESPONSE: We would like to thank reviewer 2 for highlighting this missing part. Based on renumbering of the figures, figure 2 is changed as figure 1. The sigma level for electron density map is indicated in figure 2. The text change to ‘’2*Fo*-*Fc* simulated annealing-omit map at 1 sigma level is colored in gray’’.

8) Figure 8: space missing "(C)Streptavidin…"

RESPONSE: Thanks to reviewer 2 for pointing out this mistake. We apologize for that. The legend of figure 7(due to reorganization of the figures) rearranged based on comments.

9) Supp Figure 1: in the box next to 1997, I think you meant Trp 120 and. Not Trp 20

RESPONSE: Thanks to reviewer 2 for pointing out this mistake. We apologize for that. It is corrected as ‘’Trp120’’ in the mentioned box of Supplementary Figure 2 (due to reorganization of the figures).

10) Supp Figure 1: in the box next to 2020: typo Raidation-damage free instead of Radiation-damage free

RESPONSE: Thanks to reviewer 2 for pointing out this mistake. We apologize for that. It is corrected as ‘’Radiation-damage free’’ in the mentioned box of Supplementary Figure 2 (due to reorganization of the figures).

11) Supp Figure 1: legend: you probably meant Trp120 and not 20.

RESPONSE: Thanks to reviewer 2 for pointing out this mistake. It is corrected as ‘’Trp120’’ in the mentioned box of Supplementary Figure 2 (due to reorganization of the figures).

12) Supp Figure 1: legend: add space after “2011: Relatively”

RESPONSE: Thanks to reviewer 2 for pointing out this mistake. The space was added after “2011: Relatively” in the legend of Supplementary Figure 2 (due to reorganization of the figures).

13) Supp Figure 2: add sigma level for electronic density map

RESPONSE: We would like to thank reviewer 2 for highlighting this missing part. Due to renumbering of the figures, former Supplementary figure 2 is now Supplementary figure 4 in the new version of manuscript. The sigma level for electron density map is indicated in the Supplementary Figure 4 (due to reorganization of the figures). The text changed as ‘’2*F*o-*F*c simulated annealing-omit map at 1 sigma level is colored in gray.’’

14) Supp Figure 5: add sigma level for electronic density map

RESPONSE: We would like to thank reviewer 2 for highlighting this missing part. Due to renumbering of the figures, former Supplementary figure 5 is now Supplementary figure 8 in the new version of manuscript. The sigma level for electron density map is indicated in the Supplementary Figure 8 (due to reorganization of the figures). The text changed as ‘’2*F*o-*F*c simulated annealing-omit map at 1 sigma level is colored in gray.’’

15) Supp Figure 10: authors mentioned 1Fo-Fc map. Is it a typo for 2Fo-Fc map or do you mean Fo-Fc map? Please, add sigma level as well.

RESPONSE: Thanks to reviewer 2 for pointing out this mistake. We apologize for the typo. Due to renumbering of the figures, former Supplementary figure 10 is now Supplementary figure 12 in the new version of manuscript. The mistake is corrected as 2Fo-Fc map and 1 sigma level for Supplementary Figure 12 (due to reorganization of the figures). The text changed as ‘’2*F*o-*F*c simulated annealing-omit map at 1 sigma level is colored in gray.’’

16) Supp Figure 11 and 12: are not mentioned at all in the manuscript

RESPONSE: We would like to thank reviewer 2 for highlighting this missing part. Due to renumbering of the figures, former Supplementary figure 11 is now Supplementary figure 13 and Supplementary figure 12 is now Supplementary figure 14 in the new version of manuscript. Supplementary Figure 13 and 14 are added to the main text.

17) Supp table 1: please report Rmeas and not Rmerge.

RESPONSE: The table was changed according to the reviewer's request and Rmeas is reported in the Supplementary Table 1.

18) Supp table 1: Statistic seems very poor for Cryo-Streptavidin for completeness. I would not publish a structure with 48% completeness in the high-resolution shell neither with 80% completeness overall. I would have a least 90% for the high-resolution shell and >95% overall. The CC1/2 is also quite low. You should cut off the resolution at a lower resolution to have a more acceptable completeness.

RESPONSE: We agree with our reviewer. The completeness at 1.18 Å is 86.7%. However, including the data extended to 1.1 Å improved the quality of the maps without negatively impacting Rfree and Rwork. As a result, we added a sentence at the table legend indicating the completeness at 1.18 Å.

19) Supp table 1: Could you add redundancy and completeness for the high-resolution shell for SFX data?

RESPONSE: We have updated Supplementary Table 1 with the high-resolution shell values for all reported criteria and apologize for this gross oversight in the original submission. (The error in the reported overall completeness value was due to an inaccuracy in setting the low-resolution limit when calculating the statistics, it was formerly mistakenly set to 50 Å instead of 48.49 Å. This has now also been corrected). We were not diffraction-limited for this experiment. It was, in fact, not possible to move the detector closer to the interaction region due to experimental constraints. The Wilson statistics confirmed a good fit to the data down to a resolution of 1.7 Å.

20) Supp table 1: Note 1: One crystal was used for each dataset. This is true for the synchrotron data but untrue for the SFX data.

RESPONSE: Considering the suggestions, necessary corrections were made on the table legend.

21) Supp table 2 to 6: I would have like to have the RMSD for the loop 3,4 added to the tables (maybe in parenthesis).

RESPONSE: Based on previous comments of reviewers, Supplementary table 2 to 5 merged as Supplementary Table 2 and RMSD for the L3,4 (residues 45-52) added to the Supplementary Table 2 in parenthesis.

22) To make interaction matrices more readable, I would add where the L3,4 is in the sequence number.

RESPONSE: As suggested, we created a new supp figure to show the location of L3,4 in the sequence and secondary structure.

Major comments:

1) From the manuscript, I understand there is no significant difference between the cryo- SSRL structure and the room temperature (RT) MFX structure. How can the authors justify the use of XFEL for solving the RT structure? Radiation damage should not be a main concern as there is no metal/ion in the protein. Using an XFEL for just a RT structure is an overkill as you can do it routinely in synchrotron.
2) What are the new features the SFX structure is bringing compared to the synchrotron structure or what have been done before?
3) The general feeling on this paper is that the GNM study could have been done with previously solved structure. I don't think the GNM has to use a RT structure. In molecular dynamic simulation, most of the model are issued from cryo-crystallography. For example, 1SWB and 5JD2 are also tetramer and could have been use for this study.

RESPONSE: For major comments 1, 2 and 3: We would like to thank our Referee for bringing up those issues. We agree with our Reviewer according to the data obtained by SFX. The main purpose of this study was not to get only the RT structure of streptavidin without radiation damage, which may provide the most physiologically relevant data. The femtosecond data collection speed and high precision of the MFX instrument, low mosaicity of the small crystals collectively could improve the binding site residues' electron density. This may help to provide better conformation

determination, which is crucial for ultimate GNM analysis. First, we compared the suggested ambient temperature synchrotron apo-structure of streptavidin (1SWB) with our Apo_SFX structure. 1SWB has 1.85 Å resolution, R-free 0.253, overall R-observed 0.174 while Apo_SFX structure has 1.7 Å resolution, R-free 0.2242 and R-work 0.1904. The 1SWB structure has missing residues in chain B at 45-48th positions, in chain C and D at 46-48th positions as mentioned in Supplementary Fig. 15. Moreover, 1SWB was observed with lack of electron density at residues Gln24, Lue25, Val47, Glu51, Arg53 in different chains, thus this structure was not the best model for applying GNM analysis. Moreover, for a fair comparison, we compared the electron density of binding site residues between our Apo_SFX structure and with the latest synchrotron structure of apo streptavidin (PDB ID: 3RY1), but the electron density of the loops, unfortunately, was not enhanced significantly. Overall, 3RY1 has 1.03 Å resolution, R-free 0.135 and R-work 0.117, while Apo_SFX (PDB ID: 7EK8) has lower resolution. On the other hand, in our Apo_SFX data, some of the binding site residues' electron density enhanced, and continuous electron density without alternate side chains conformations was observed. Chain A at Apo_SFX structure between Ile30-Thr40, Ala46-Arg53, and Glu44 have better electron density and precise side-chain conformations compared to the 3RY1 structure as indicated in the following figure. In chain B, between Asn23-Gly26, Phe29-Leu39, Ser45-Gly48, Glu51-Val55, and Thr42 there is more precise electron density and lack of alternate side-chain conformations for Apo_SFX structure. In chain C Asn23-Leu25, Ile30, The32, Ala35, Glu44, and Glu51 have better and continuous electron density with less alternate conformations at Apo_SFX structure, however between Ala46-Ala50 and Arg53, the electron density is better at 3RY1, but similar conformation with Apo_SFX. Similarly, in Chain D Apo_SFX structure has a better density and precise conformations for Asn23-Leu25, Ile30, The32, Ala35-Ala38 (but not Asp36), and Thr42, however, for Glu44-Ser52 3RY1 structure has better electron density but similar conformation with Apo_SFX. The following figure was also added as Supplementary Fig. 16. The findings about 1SWB and 3RY1 comparison was also added in the result section. On the other hand, we would like to compare the closest data in GNM analysis. For this purpose the new Apo_SFX structure and our previous 5JD2 structure have the same X-ray source and RT data collection properties, that's why we minimized the temperature and device relevant differences and artifacts with those structures. This part was also mentioned at the end of the introduction section as "The new Apo_SFX structure, which has better resolution and electron density from previous ambient temperature apo-structures, and 5JD2 have the same X-ray source and ambient temperature data collection properties, which minimize the temperature and device relevant differences and artifacts with those structures for a proper comparison in GNM analysis." Moreover, as mentioned in the materials and methods section "For a better comparison for GNM analysis, 5JD2 and Apo_SFX crystals were obtained from the same batch with minimized artifacts such as crystallization conditions, mother liquor and protein sample."

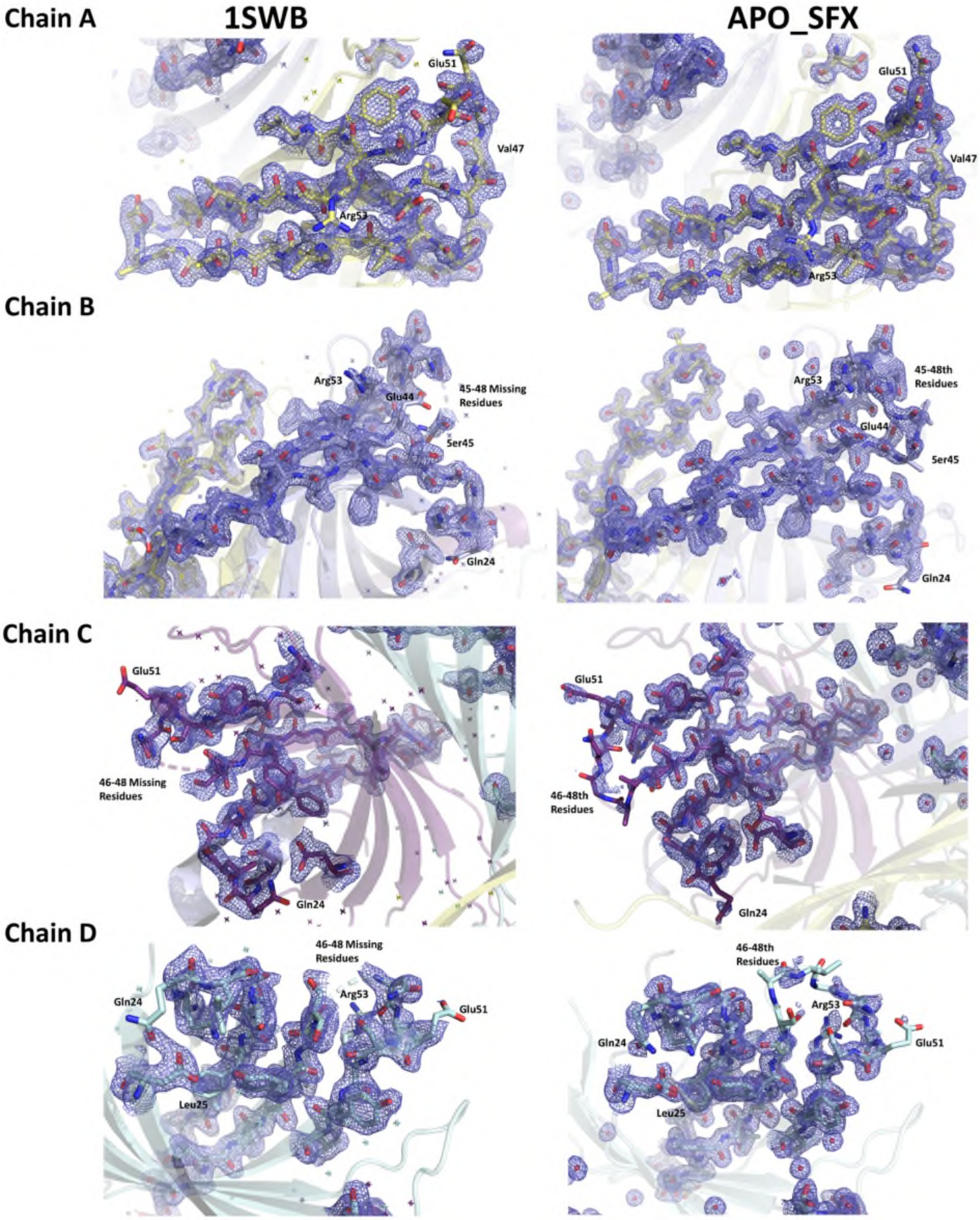

The figure was indicated as Supplementary Fig. 15

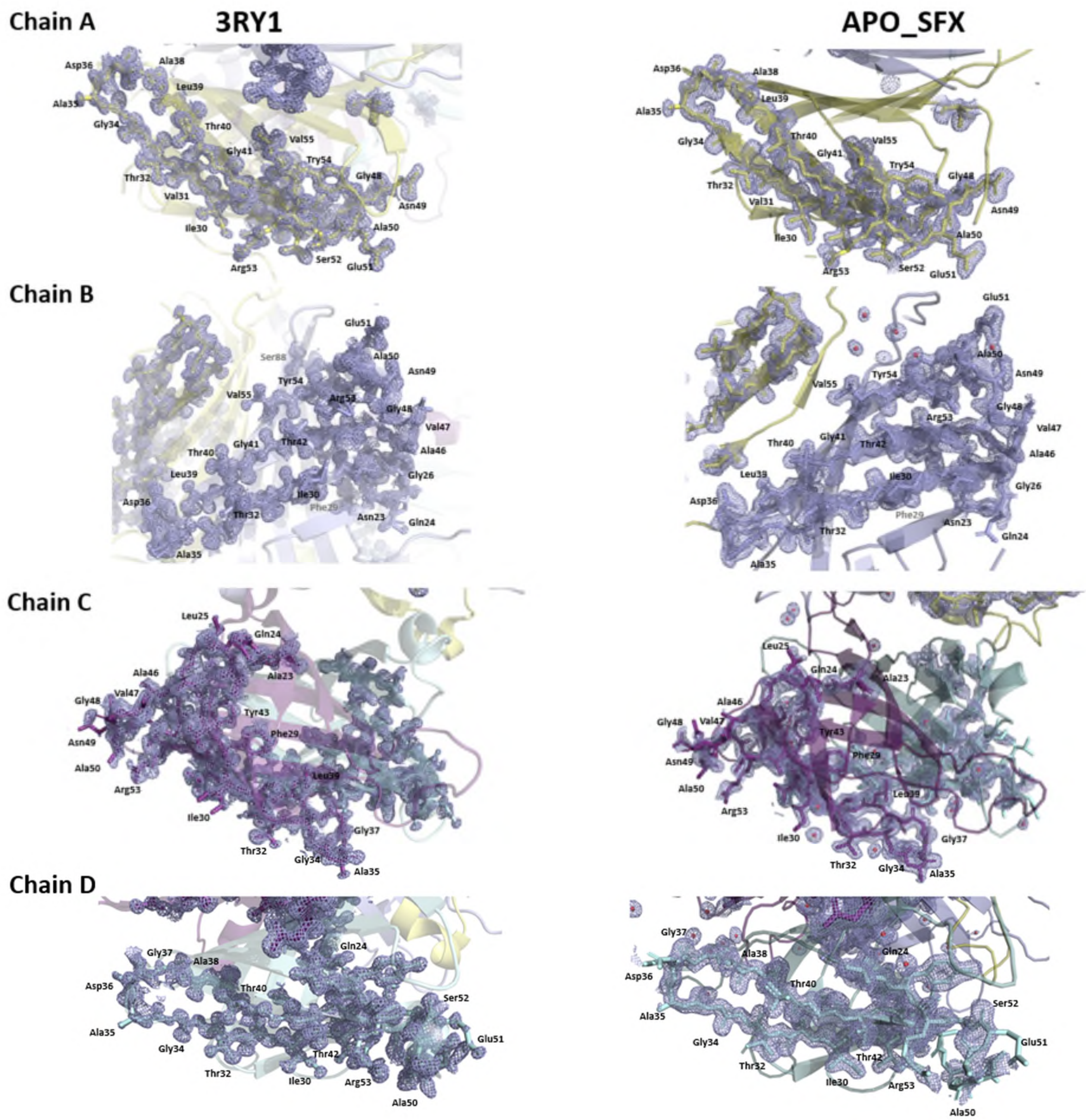

The figure was indicated as Supplementary Fig. 16.

In conclusion, the novelty of this paper does not reside into the structures as they are very similar to previous ones and don't seem to bring anything new, but in the GNM analysis. The GNM analysis shows the importance of key residues in the mechanism of cooperative allostery. To confirm hypothesis from GNM, the authors suggest at the end of the discussion a time-resolved study in the future. This kind of study will definitely justify a serial crystallography approach.

****

Reviewer #3 (Remarks to the Author): General Remarks: Streptavidin is a very important biotechnological tool, being able to capture biotin and biotin derivatives with affinities higher

than other commonly used capturing molecules, i.e. antibodies, aptamers, etc. Albeit engineered derivatives were reported to work as monomers or dimers, the wild-type molecule, that coordinates four streptavidin monomers, holds the highest affinity. However, full occupancy of all four binding sites is still debated. The quaternary structure was solved several times, at varying resolutions, with different ligands, and by different approaches, x-ray crystallography, and later by cryoEM. Streptavidin can be considered a model system to study how multimeric proteins exert their function and how each monomer influences the others is still a focus of study, intrinsically challenging. In streptavidin, Trp120 appears to be important in the intradimer allostery. Ligand binding uses loop 3/4 appears to function as a “lid”, closing over the binding pocket when biotin-bound. However, in the apo-state L3/4 appears very flexible and many times unsolved. How the “lid” opening and closing propagates to the binding pocket and how this propagates to the neighboring monomer through trp120 remains under study. The present study tackles the above questions using the latest systems to resolve crystal structures at ambient temperatures. This new approach is providing novel conformational states at atomic resolution of several proteins and also of highly complex systems as the 30S ribosome. The authors take a further step and solve the same apo structure using classical x-ray diffraction at cryogenic conditions. In addition, the authors compare their new structures to previously reported structures of streptavidin complexed with seleno-biotin/biotin or other apo states. Ayan et al. describe defined electron densities for L3/4 for the Apo structures, at both, ambient and cryogenic conditions. Interestingly, not all L3/4 loops appeared in the open state. 3 out of 4 lids appeared open while one was similar to biotin-bound closed lid. A comparison with the seleno-biotin bound structure, also obtained at ambient temperature, indicates a certain asymmetry among monomers. In the bound state, 3 out 4 lids were closed while one stayed open. Another interesting finding relies on the intramonomer conformational coupling between the lid and the binding site. For instance, the density of positive charges in the binding pocket decreases if the lid is closed, similarly to when biotin is bound. This indicates that redistribution of charges in the binding pocket is not mediated by biotin. Also, coordinated water molecules are affected, yet more dependent on biotin binding rather than the lid state. In general, the coordinated number of water molecules appears reduced when biotin bound. The Gaussian Network Model analysis highlights several intra- and interchain correlated conformational changes.Altogether, the work of the DeMirci group provides new results and analysis defining how the tetrameric streptavidin orchestrates to capture biotin. The work is technically well executed by an expert team. Nevertheless, the manuscript requires a number of adjustments, here you can find some suggestions:

Major concerns:

1) The main goal and objectives of the study are not clearly introduced to the reader. Also, the reason behind the necessity to solve the structure at ambient or physiological temperatures is not clear, what is expected? How is this approach

providing new insights into other complexes/proteins? Why is it important to solve the Apo state? Introducing the reader to the above can tremendously increase the clarity of the manuscript.

RESPONSE: Cryogenic temperatures can introduce bias for structure determination as cryogenic temperatures may disrupt the overall protein backbone fold. The crystallographic data without radiation damage at ambient temperature provides better structure determination from small micro crystals by using ultrabright X-ray sources. Moreover, there are unsolved residues which are especially observed in the loop region and emphasized in the main text (discussion part in the last paragraph) as ''Ligand binding uses loop 3/4 appears to function as a "lid", closing over the binding pocket when biotin-bound. In the apo-state L3/4 appears very flexible and many times unsolved''. Moreover, the conformational changes in the binding pocket with and without ligand play a key role in the engineering of new biological tools using the streptavidin-biotin system. Ultrafast data collection speed and high precision of the MFX instrument could enhance this binding site residues' electron density, which provide better conformation determination, which is crucial for ultimate GNM analysis. Thus, while we were aware that there was an ambient temperature Apo-state structure of streptavidin (1SWB), we performed SFX for apo_streptavidin. 1SWB structure has missing amino acid residues at the crucial 3/4 loop at binding site (described in new supplementary fig. 15), however our Apo_SFX structure provides those residues and other missing ones with much improved electron density and resolution. As it is mentioned in our manuscript (in introduction section), "The new Apo_SFX structure, which has better resolution and electron density from previous ambient temperature structures, and holo 5JD2 structure have the same X-ray source and ambient temperature data collection properties, which minimized the temperature and device relevant differences and artifacts with those structures for a proper comparison in GNM analysis". By determining the ambient temperature apo-state streptavidin structure, we would like to have the closest template to compare ligand-bound state and apo-state comparison. For this purpose, "5JD2 and Apo_SFX crystals were obtained from the same batch with minimized artifacts such as crystallization conditions, mother liquor and protein sample." as newly mentioned in the materials and methods section.. This data was compared with the ligand-bound structure in GNM analysis to provide a better understanding of the effect of the ligand on the structure dynamics. Lastly, the indicated text (discussion part in the last paragraph) is added to the main text to emphasize the main goal of this study ''To determine and validate the accuracy of previous structures of streptavidin, SFX offers structural data without temperature and radiation side effects, leading to a solid template for future studies. The next step to better understand the details of this binding and cooperative allosterism is performing time-resolved structural analysis by using ultrabright and ultrafast XFELs.'' at the end of the discussion section. Also, "We re-evaluated the tetrameric structure of streptavidin by Gaussian Network Model

(GNM) analysis of the protein's dynamics [26] by inspecting the Apo-SFX and selenobiotin-bound streptavidin (PDB ID: 5JD2) structures. .... To understand and confirm those dynamics, we investigated polar interaction network, number of coordinated water molecules, thermal ellipsoid structure and electrostatic surface of L3/4 loop for ligand bound and APO-SFX structures." was added in the introduction section to clear the purpose of this study.

2) The results section appears very slim. Seven main figures and seven supplementary figures are packed into two pages of text. This section could be extended with better descriptions of the main findings. Now they appear hidden within other less important results or comments. For example: in the 2nd paragraph, "We observed additional electron density that belongs to the residues in the L3,4 region which were not modeled in previous studies". This deserves a more detailed description of the result. Here go some examples where the authors could expand and explain better:

RESPONSE 2 and 3: We would like to thank the referee for this issue. We explained the minor and major differences in more detail with Supplementary Fig. 15 and 16 and in the result section as "First we compared the ambient temperature synchrotron apo-structure of streptavidin (1SWB) with our Apo_SFX structure. 1SWB has 1.85 Å resolution, R-free 0.253 without R-free while Apo_SFX structure has 1.7 Å resolution, R-free 0.2242 and R-work 0.1904. The 1SWB structure has missing residues in chain B at 45-48th positions, in chain C and D at 46-48th positions as mentioned in Supplementary Fig. 15. Moreover, 1SWB was observed with lack of electron density at residues Gln24, Lue25, Val47, Glu51, Arg53 in different chains, thus this structure was not suitable for applying GNM analysis. Moreover, for a fair comparison, we compared electron density of binding site residues between our Apo_SFX structure and with the more recent synchrotron structure of apo streptavidin (PDB ID: 3RY1), but electron density of the loops, unfortunately, was not enhanced significantly. Overall, 3RY1 has 1.03 Å resolution, R-free 0.135 and R-work 0.117, while Apo_SFX (PDB ID: 7EK8) has 1.7 Å resolution, R-free 0.2242 and R-work 0.1904. On the other hand, in our Apo_SFX data, some of the binding site residues' electron density enhanced, and continuous electron density without alternate side chains conformations was observed. In particular, chain A at Apo_SFX structure between Ile30-Thr40, Ala46-Arg53, and Glu44 have better electron density and precise side-chain conformations compared to the 3RY1 structure as indicated in the following figure. In chain B, between Asn23-Gly26, Phe29-Leu39, Ser45-Gly48, Glu51-Val55, and Thr42 there is more precise electron density and lack of alternate side-chain conformations for Apo_SFX structure. In chain C Asn23-Leu25, Ile30, The32, Ala35, Glu44, and Glu51 have better and continuous electron density with less alternate conformations at Apo_SFX structure, however between Ala46-Ala50 and Arg53, the electron density is better at 3RY1, but similar conformation with Apo_SFX. Similarly, in Chain D Apo_SFX structure has a better density and precise conformations for Asn23-Leu25, Ile30, The32, Ala35-Ala38 (but not

Asp36) and Thr42, however, for Glu44-Ser52 3RY1 structure has better electron density but similar conformation with Apo_SFX.”

3) Page 5: “… we observed the minor conformational changes ...” The authors do not define or mention how many As imply minor of major conformational changes.

RESPONSE: The manuscript has been changed with “we observed the minor conformational changes which are less than 1 Å at the residues…” for clearing this issue.

4) Page 7: “Slowest 10 modes reveals the globul residue motions”, I am not sure if the authors meant “global” or “globular”.

RESPONSE: Thanks to the referee for pointing out this mistake. We apologize for the typo. We fixed it as global.

5) Page 8: “These results confirm biotin binding may not have a stabilization effect however contribute to the allostery of streptavidin” This is not clear,the authors may need to rephrase this sentence. Allostery is a property of certain proteins, then, it is not clear how the biotin binding might contribute to the allostery of streptavidin. Ligands can cause allosteric responses, they do not contribute to allostery.

RESPONSE: Thanks to the referee for pointing out this mistake. We fixed it as “These results confirm biotin binding may not have a stabilization effect however can cause the allosteric response of streptavidin”

6) Page 9: “However, our atomic resolution structural and GNM data suggest that there is a predisposed cooperative allosterism before binding of the first biotin molecule.” This sentence is not clear, do authors mean that “predisposed cooperative allosterism” is a property of the streptavidin? Or do they imply the protein has a certain predisposition to bind, particularly, biotin with cooperative allosterism?

RESPONSE: Thanks to the referee for pointing out this mistake. We fixed it as “However, our atomic resolution structural and GNM analysis suggest that there is a predisposed property of streptavidin cooperative allosterism before binding of the first biotin molecule.”

7) In general, the results segment could be described in more detail, likely considering rearranging some of the main figures. A good rule of thumb is that each figure should relate to a results segment, supporting a well described finding.

RESPONSE: We believe that our article has made good progress thanks to the pragmatic comments and suggestions of our reviewers. We tried to address all comments and suggestions, the organization and layout of the figures have been changed and also the results segment detailed.

8) Figure 1 does not add to the manuscript since the novel approach is not the main focus of the paper. Perhaps, Figure 1 could go to supplementary figures. An alternative would be to combine figure 1 with a scheme of the study indicating the main question to be addressed

RESPONSE: We are grateful for this recommendation from our referee. We moved Fig 1 to the Supplementary figures.

9) Figure 4: comparison of the SFX structure with the apo structure obtained by cryoEM, is it necessary in the main figures? It is not used for any other further comparison (i.e. Fig. 6, Fig. 8, Fig. 9). Perhaps it could be moved to supplementary figures.

RESPONSE: We would like to thank our referee for this recommendation. As suggested we moved the figure to the Supplementary figures as Sup Fig. 5.

10) Supp Fig 9 adds interesting information which together with Fig 6, and Fig 8 could become a main figure summarizing how the binding pocket topography, charges, elasticity and water coordination changes as a function of, for example, lid state, or biotin-bound to unbound.

RESPONSE: We would like to thank our referee for this recommendation. We moved Sup Fig 9 to the main text.

Scientific:

11) I remained puzzled about the similarities between the ambient and cryo structures. What defines the conformational state of streptavidin? How much the crystallization process is influencing? What is the contribution of the increased temperature?

RESPONSE: Minor conformational differences on the residues in the active site (the residues 23, 27, 45, 49, 88) are observed between the ambient and cryo structure (Fig. 2). These two structures come from the same crystallization condition (Pact Premier™ 100 mM MMT buffer pH 6.0 and 25 % w/v PEG 1500) and have the same space groups (P 1 21 1). The similar cell dimensions indicated below and Supplementary Table 1.
a, b, c (Å)

sfx: 47.40, 87.70, 58.90
cryo: 46.36, 85.76, 58.13
α, β, γ (°)
sfx: 90.00, 98.90, 90.00
cryo: 90.00, 98.71, 90.00
However, we perform SFX to offer a more accurate model to minimize the temperature and device relevant differences and artifacts with those structures for a proper comparison in GNM analysis with the same X-ray source and ambient temperature data collection properties in this paper. Moreover, "5JD2 and Apo_SFX crystals were obtained from the same batch with minimized artifacts such as crystallization conditions, mother liquor and protein sample." to minimize the sample preparation bias as newly mentioned in the materials and methods section.

12) How do the new apo structures compare to other Apo-states that had been resolved previously? The comparison with PDB: 6J6K, obtained by cryoEM, shows different conformations of L3/4, all closed albeit obtained the Apo state (Fig. 4), how do you interpret this?

RESPONSE: We would like to thank the referee for this question. Fan et al. (6J6K) structure focuses more on the innovation and improvement of single particle cryo-EM reconstruction. They couldn't observe any electron density for this loop.

Fan et al. discussed this issue below:
''Compared with the biotin-bound SA, the density corresponding to loop 46–51 in the EM map of apo-SA was missing (Fig. 2), indicating that this lid-like loop is flexible without ligand binding.''
''Fan, X., Wang, J., Zhang, X. et al. Single particle cryo-EM reconstruction of 52 kDa streptavidin at 3.2 Angstrom resolution. Nat Commun 10, 2386 (2019). https://doi.org/10.1038/s41467-019-10368-w''

13) Page 9: "Together with observed conformational changes in the biotin binding pocket (Fig 5), apo streptavidin (PDB ID: 6J6K) obtained by CryoEM was superposed with our SFX structure (Supplementary Fig 3). The comparison of the structures suggests that the two techniques can capture alternative binding conformations and expand the conformational space sampling of the active site loop." Taking into account that the RMSD between the binding site residues of apo and holo structures is less than 0.4A, how can the authors assure these differences are only usual fluctuations of the residues, or error during the experimental elucidation of the structures, or they are actually real differences between the apo and holo states of streptavidin?

RESPONSE: We would like to thank the reviewer for his/her comment. The RMSD was calculated for the overall structure, now we added the RMSD for the loop to provide a more detailed and clear expression of differences in the Supplementary Table 2 in the parenthesis. The major differences between 6J6K and our structure is generated because of the loop region (residues 42-52). We suggested that ligand binding using loop 3/4 appears to function as a "lid", closing over the binding pocket when biotin-bound. The RMSD for the L3,4 is overall 3.572 (for chain A: 2.879, chain B: 4.484 chain C: 3.276 , chain D: 3.649) , leading to a major difference.

14) During the GNM analysis, the authors mention they used the 10 slowest modes to be correlated with the global motions of the protein, and the 10 fastest modes for localized motions. So, how many modes were in total calculated by the GNM analysis?

RESPONSE: In GNM analysis for the N number of atoms selected, N-1 number of non-zero modes can be calculated. Thus, in this study, the selected atom number in 5JD2 structure with selenobiotin was 489; and 488 non-zero modes were calculated with GNM. Similarly, the selected atom number in the Apo-SFX structure is 476 (all carbon alpha atoms); and 475 non-zero modes were calculated with GNM. Also, the selected 10 slowest modes corresponded to the 10 modes with the smallest eigenvalue (high variance) and the 10 fastest modes correspond to the 10 modes with the highest eigenvalue (low variance). We show the high variance (bigger than 1) in the slowest 10 modes below. Furthermore, we had decided to only focus on the slow modes, so we removed any mention of the fast modes. We also rewrote the method section to provide the correct details and increase the reproducibility of the analysis.

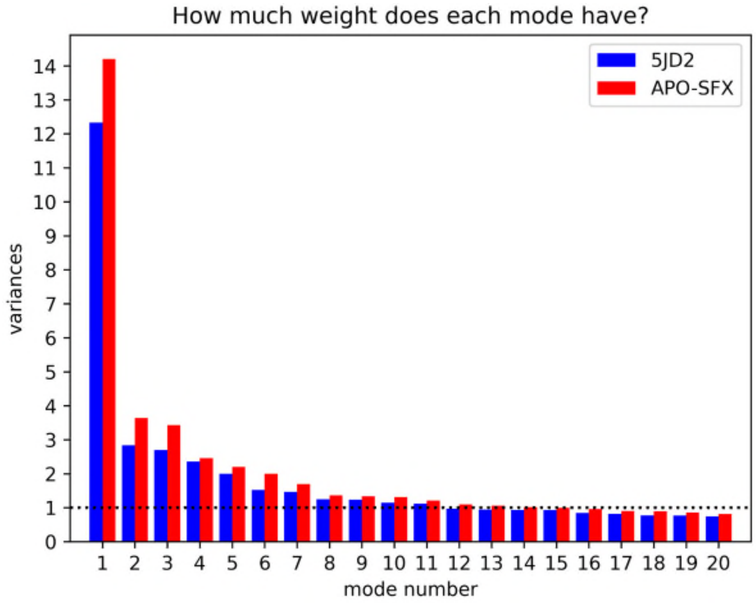

15) Supplementary Figure 1 refers to structures obtained in “2020”, intended to the structures of this work. Please update to 2021 and indicate that they belong to “this work”. Also, “2014” in the captions should be bold to maintain consistency.

RESPONSE: Thank you for this recommendation of our referee. Considering the suggestions, necessary corrections were made to the figure and legend.

16) The units of the KD is missing in 4th line of the second paragraph of the introduction. Should read: KD = 10-14 to 10-15 M

RESPONSE: Thanks to the referee for pointing out this mistake. We fixed that as Kd = 10^-13 to 10^-15

17) Reference 10 suggests that modifications in biotin may cause a “more “disordered” L 3/4 loop in avidin, rather than in streptavidin. Although both are highly conserved, there are differences, specially in L 3/4. Please, indicate what is the important role of L3/4 mentioned in the last sentence of the second paragraph of the introduction.

RESPONSE: Thanks to the referee for pointing out this missing part. We expanded the part as shown below. ‘’Protein regions which are involved in protein-protein and protein-target interactions have high plasticity and are often highly flexible. In addition, these regions are usually located on the solvent exposed surface of protein. L3,4 is the crucial part of streptavidin that interacts with biotin, regulate the binding and plays a primary role in binding.’’ As suggested, we create a supplementary figure 1 to show the location of L3,4 in the sequence and secondary structure.

18) Nomenclature of the different structures analyzed: please keep consistency, specially related to the SFX (ambient) structures. Sometimes it is called “ambient structure”, “SFX structure” “APO-SFX”, and so on.

RESPONSE: We fixed all as Apo-SFX.

19) Bound not bounded, correct along the text.

RESPONSE: We fixed the text as “bound”.

REVIEWERS' COMMENTS:

Reviewer #1 (Remarks to the Author):

This paper could be a nice paper. However, there are grammatical mistakes, logical inconsistencies and odd expressions that make a review difficult if not impossible. This reviewer is saddened by the lack of attention to detail. This reviewer already pointed out numerous odd expressions in the previous version. The present version is not better. In order to make this publishable this reviewer urges the corresponding author, who spent most of his career in the US, to correct the text throughout and pay attention to expression and logical flaws. A number of examples are listed below. Please make sure that the paper is prepared to professional standard.

odd sentence, please correct. How can structures have the same X-ray source? (maybe: structures are determined at the same X-ray source, or structures are determined from data collected at the same X-ray source).

108 The new Apo_SFX structure, which has better resolution
109 and electron density from previous ambient temperature apo-structures, and 5JD2 have the same
110 X-ray source

odd sentence, please correct:

112 To understand and confirm those dynamics, we investigated polar
113 interaction network, number of coordinated water molecules, thermal ellipsoid structure and
114 electrostatic surface of L3/4 loop for ligand bound and APO-SFX structures

odd expression, how can a structure have resolution? (a structure is determined at … resolution).
140 1SWB has 1.85 Å resolution,
141 R-free 0.253 without R-free while Apo_SFX structure has 1.7 Å resolution, R-free 0.2242 and R
142 work 0.1904.

l.153 ff: chain A HAS

l. 212. the authors mention microstate. Define microstate.

l. 267, odd sentence: The weighted 10 slowest mode results which reveal the protein’s global motions are provided in Fig. 8E.

l. 325 ff …, please correct grammatical errors …, articles are missing, expression.

l 361. expression, two verbs “uses” and “appears”.
361 Ligand binding uses loop 3/4 appears to
362 function as a “lid”, closing over the binding pocket when biotin-bound.

l. 424: what is SFX evaluation?

l. 447 ff. Gibberish. Please get yourself a recent SFX paper and read how to properly describe hit finding and indexing.

l. 448. The authors admit in the rebuttal letter that they used phenix for thermal ellipsoid generation. This is not reflected here.

l. 495. what is a Kirchhoff matrix?

Fig. 3. Poor expression in the figure caption, please correct

Fig. 5 Poor expression in the figure caption, please correct.

Fig. 6. Delete ‘based on B-factor’

Reviewer #2 (Remarks to the Author):

The changes the authors made to the manuscript improved both reading and clarity. I appreciated the clear answers in the rebuttal letter for all the different concerns raised by the reviewers. The introduction on GNM analysis is much better. The overall explanation the authors gave about the necessity for Apo-SFX structure is satisfactory.
The second version of the manuscript is much better.

I still have few minor remarks:
1) I disagree with part of the answer in the rebuttal letter from my minor remark nº5. I was just curious about the criteria applied with CrystFEL to determine the resolution cut-off. I understand it is based on CC* which is quite specific for CrystFEL and XDS. I disagree with the sentence “Resolution cut-off for all crystallography data is generally based on CC* and not CC1/2.” Different programs use different parameters, e.g. Dials uses CC1/2. Also, CC* is based on CC1/2.
2) Line 77: I don’t understand the analogy to “plug and play” type of molecule
3) Line 92-94: I agree with the authors about the fact that “cryogenic temperature can introduce bias”. Could the author backup this statement with reference(s)?
4) Lines 114, 280, 314, 361, 362, sup Fig 2 and 6…: Could you please either call in the manuscript the loop L3,4 or L3/4 loop?
5) Line 141: I don’t understand “R-free 0.253 without R-free…”
6) Line 225: what is the cut-off distance? I suppose it is the cut-off for the correlation between residues. Why do you have 2 different cut-off distances? What is the unit? Å?
7) Line 307-309: Just being curious… You should have enough structural data to build an hybrid tetramer with each dimer having only one biotin bound. GNM studies on this tetramer may answer your hypothesis. Maybe for another paper...
8) Line 458: Replace (by 28%
9) Line 465: I would add here the answer you gave me for my remark nº18 about the low completeness (~80%) in the high-resolution shell for the cryo structure.
10) Line 469: as you put the instrument used at LCLS, I would add the instrument BL-12-2 as well for SSRL.
11) Figure 4E: I don’t really understand which one is 5JD2 and which one is Apo-SFX. In blue, you have close conformation so it should be 5JD2 and in red open conformation then Apo-SFX. But chain A from Apo-SFX is in closed conformation, hence my confusion for the Chain A in figure 4E.
12) Supplementary Fig. 1: What is the difference between green arrow and red dot? And what is the red arrow?
13) Supplementary Fig. 5: Yellow text is hard to read
14) Supplementary Fig. 11: Black square are not described in the legend
15) Supplementary Table 1: replace LCLS by LCLS (MFX) and you could change Beamline by Instrument if you like.
16) Supplementary Table 1: I am still missing Rmeas for the cryo structure.

Reviewer #3 (Remarks to the Author):

The manuscript by Ayan et al. has increased in clarity from the previous version. Most concerns have been addressed. The novel Apo-SFX structure provides enhanced details of the tetrameric streptavidin, several segments and side chains are resolved continuously. Particularly, the 3,4 loop (L3,4) of each monomer is clearly resolved. The authors use GNM to explore the correlated movements of residues on each monomer as part of the Apo-SFX and compared to the biotin bound structure, also solved at ambient temperatures (PDB: 5JD2). However, the correlation between residues in L3,4 and others in the binding site, dimer interface, or dimer/dimer interaction appear to be unnoticed by GNM or they are absent. This, in turn, would indicate that the "Lid" movement does not propagate to other parts of the complex or it is not influenced by the rest of the complex. Nevertheless, the Apo-SFX structure shows that L3,4 is closed in one monomer out of three. The opposite occurs in the Holo-SFX structure, where one monomer shows an open lid albeit being bound to biotin. The authors and others interpret these findings as L3,4 being intrinsically flexible. However, this can be also explained by coordinated allosterism. In this case, the GNM should have evidenced such correlations.

Altogether, the work by the DeMirci group provides new insights into the functioning of the multimeric streptavidin.

Here, you can find several issues that were annotated during this revision.

Minor issues:

Revise the order of supplementary figures. They do not appear orderly in the text.

Lines 65-67: "L3,4…. Is the crucial part of streptavidin that interacts with biotin and regulates the binding".
References required. However, whether L3,4 loop regulates biotin binding or not may be overstated. Please, revise also the following sentence, appears redundant.

Lines 92-94: " However, the available apo-state structures are still limited as cryogenic temperatures can introduce bias for structure determination by disrupting the overall protein backbone fold."
Review this sentence. First, there are apo-state structures available. Second, what is the evidence available for structural biases introduced by cryogenic temperatures?? Need some references here.

Line 108: "Apo_SFX" vs "Apo-SFX". Correct along the MS for consistency. Also, I suggest to use Biotin-SFX or Holo-SFX instead of (5JD2) for clarity.

Lines 139-140: "First we compared the ambient temperature synchrotron apo-structure of streptavidin (1SWB) with our Apo_SFX structure"
Reference is required for PDB 1SWB.

Line 147: a reference for 3RY1 is required.
Line 150: PDB 7EK8?

Lines 171-173: "…..biotin substrate. Interestingly, this conformational change is more prominent for chains B, C, and D."
This sentence appears unclear, does it relate to Apo_SFX or to 6J6K?. Refer to a figure that shows these conformational changes.

Line 183: review the use of "determined", likely you mean "found"

Line 280: “3/4 loop” should read

REVIEWERS' COMMENTS:

Reviewer #1 (Remarks to the Author):

This paper could be a nice paper. However, there are grammatical mistakes, logical inconsistencies and odd expressions that make a review difficult if not impossible. This reviewer is saddened by the lack of attention to detail. This reviewer already pointed out numerous odd expressions in the previous version. The present version is not better. In order to make this publishable this reviewer urges the corresponding author, who spent most of his career in the US, to correct the text throughout and pay attention to expression and logical flaws. A number of examples are listed below. Please make sure that the paper is prepared to professional standard. ✓✓✓✓

We sincerely apologize for all the typos and other issues. We extensively reviewed our manuscript and fixed all the existing points raised by our Reviewer 1.

1. odd sentence, please correct. How can structures have the same X-ray source? (maybe: structures are determined at the same X-ray source, or structures are determined from data collected at the same X-ray source). ✓✓✓✓

108 The new Apo_SFX structure, which has better resolution
109 and electron density from previous ambient temperature apo-structures, and 5JD2 have the same
110 X-ray source

We would like to thank Reviewer #1 for the correction. Changed "The new Apo-SFX structure, which has better resolution and electron density from previous ambient temperature apo-structures, and 5JD2 have the same X-ray source and ambient temperature data collection properties, which minimize the temperature and device relevant differences and artifacts with those structures for a proper comparison in GNM analysis." to **"The new Apo-SFX structure, which has higher resolution and improved electron density quality compared to previous ambient temperature apo-structures, and Holo-SFX structure were determined from data collected at the same X-ray source. Thus, temperature- and instrument-related differences and variables are minimized within these structures for accurate comparison in GNM analysis "**

2. odd sentence, please correct:✓✓✓✓

112 To understand and confirm those dynamics, we investigated polar
113 interaction network, number of coordinated water molecules, thermal ellipsoid structure and
114 electrostatic surface of L3/4 loop for ligand bound and APO-SFX structures

We would like to thank Reviewer #1 for his/her comments. The indicated odd sentence in the end of the introduction has been corrected to **''To highlight the structural dynamics of**

streptavidin, we investigated the polar interaction network, number of coordinated water molecules, thermal ellipsoid structures and electrostatic surface models for both ambient and cryogenic structures. Our data presented here provide a novel cooperative allosteric model for streptavidin biotin interactions.''

3. odd expression, how can a structure have resolution? (a structure is determined at … resolution). ✓✓✓✓

140 1SWB has 1.85 Å resolution,
141 R-free 0.253 without R-free while Apo_SFX structure has 1.7 Å resolution, R-free 0.2242 and R
142 work 0.1904.

We would like to thank Reviewer #1. Changed "1SWB has 1.85 Å resolution, R-free 0.253 without R-free while Apo_SFX structure has 1.7 Å resolution, R-free 0.2242 and R-work 0.1904." to **"1SWB was determined at 1.85 Å resolution with an R_{work} of 0.17 and R_{free} of 0.25 while the Apo-SFX structure was determined at 1.7 Å resolution with an R_{work} of 0.19 and R_{free} of 0.22."**

4. l.153 ff: chain A HAS ✓✓✓✓

Changed "A has disrupted symmetry and displayed an alternate conformation with a closed state similar to the selenobiotin-bound conformation" to **"On the other hand, chain A displayed disrupted symmetry and an alternate conformation with a closed state similar to the Holo-SFX conformation (Fig. 4)."**

5. l. 212. the authors mention microstate. Define microstate. ✓✓✓✓

These are the states that a protein in its native state can access by thermal fluctuations. We would like to enhance the meaning by changing the text as **"The normal modes obtained describe the microstates accessible to the protein's native state. The theoretical fluctuations calculated with normal modes from GNM correlate with the thermal fluctuations found in X-ray experimentation as well. Slow modes have the highest mode weights and contain the most collective residue motions. These are the intrinsic fluctuations required for the protein's global motion [37]."**

6. l. 267, odd sentence: The weighted 10 slowest mode results which reveal the protein's global motions are provided in Fig. 8E. ✓✓✓✓

If we explain the method in more detail; each normal mode has an eigenvector (containing residue fluctuations) and an eigenvalue (inverse gives the weight of the mode). Since the mean squared fluctuations over a mode-set are calculated by taking the weights of each mode into account, we referred to the result as "weighted 10 slowest mode". Our reason for selecting them was that they "reveal the protein's global motions". However, we realize the meaning can be better conveyed with

the changed sentence(s) as follows: **“Specifically, the mean squared fluctuations of the 10 slowest modes were obtained (Fig. 8E). These fluctuations reveal the protein’s global motion.”**

7. l. 325 ff …, please correct grammatical errors …, articles are missing, expression. ✓✓✓✓

Thank you for your comment. In the sentence “As a driving force, the closing of the lid can be caused by the allosteric and cooperative “pump-like” motion of tetrameric streptavidin for removing water molecules from the ligand binding site.” We would like to share our novel hypothesis of the “pump-like” motion which may occur by allosteric and cooperative protein dynamics. Thus, this sentence does not include any article or references. Moreover, we corrected grammatical issues and sentence structure as **“As a driving force, the closing of the lid may be caused by the allosteric and cooperative “pump-like” motions of tetrameric streptavidin which forcibly removes water molecules from the ligand binding site”**

8. l 361. expression, two verbs “uses” and “appears”.
361 Ligand binding uses loop 3/4 appears to
362 function as a “lid”, closing over the binding pocket when biotin-bound. ✓✓✓✓

Thank you Reviewer #1 for your feedback. Changed “Ligand binding uses loop 3/4 appears to function as a “lid”, closing over the binding pocket when biotin-bound.” to **“Ligand binding is characterized by loop 3/4. It functions as a “lid” that closes the binding pocket when it binds to biotin.”**

9. l. 424: what is SFX evaluation? ✓✓✓✓

Thank you Reviewer#1 for this question. SFX evaluation means data collection and analysis for SFX. The wrong part has been changed to **"SFX data was collected during the LCLS beamtime (ID: mfxp17318) at the MFX instrument of LCLS at SLAC National Accelerator Laboratory (Menlo Park, CA)”**

10. l. 447 ff. Gibberish. Please get yourself a recent SFX paper and read how to properly describe hit finding and indexing. ✓✓✓✓

Thank you Reviewer#1 for this comment. We tried to fix it by dealing with the wrong parts as follows:
“The diffraction data for the SFX structure were collected through the MFX instrument using ePix10k2M detector. Total diffraction patterns were selected as potential crystal hits using *CHEETAH* software [55]. The hit finding, which is based on Bragg reflections, was performed by using peakfinder8 and the images containing more than 20 peaks were classified as crystal hits that were indexed by using the *CrystFEL*

software package [56,57] version 0.9.0 [58]. While *XGANDALF* [59], *DIRAX* [60], *MOSFLM* [61] and *XDS* [62] were used as indexing algorithms, the indexed reflections were subsequently integrated and merged using *PARTIALATOR* [63] applying the unity model over 3 iterations and the max-ADU set to 7500. The complete reflection intensity list from *CrystFEL* was then scaled and cut using the *TRUNCATE* program from the *CCP4* suite [64] prior to further processing. For streptavidin crystals, the final dataset included 384,250 hits with a total of 106,021 indexed patterns (28%) was merged into a final dataset ($P12_11$, unit cell: a = 47.40 Å, b = 87.70 Å, c = 58.90 Å; α = 90.00, β = 98.90, γ = 90.00) (Supplementary Table 1). Additionally, using a resolution cutoff at 1.7 Å, an R_{split} of 11.0% was obtained along with a CC* of 0.99 over the entire resolution range. The final dataset had an R_{split} of 77.8%, and CC* of 0.87 in the highest resolution shell. For cryo-synchrotron structure, X-ray diffraction data ($P12_11$, unit cell: a = 46.36 Å, b = 85.76 Å, c = 58.13 Å; α = 90.00, β = 98.71, γ = 90.00),were collected by using a Dectris Pilatus 6M detector installed at BL-12-2 instrument at SSRL, which was processed with *XDS* [62] package for indexing and scaled by using *XSCALE* [62]. The resolution cutoff set to 1.1 Å without negatively impacting R_{free} and R_{work} (Supplementary Table 1).”

11. l. 448. The authors admit in the rebuttal letter that they used phenix for thermal ellipsoid generation. This is not reflected here. ✓✓✓✓

Thank you Reviewer #1 for comment. Structure refinement was done via PHENIX by using TLS parameters, leading to the visualization of ellipsoid structure via PyMOL. The explanation is added to the method section which is titled as ‘Temperature factor analysis and generation of ellipsoids’. The added sentence is indicated below with related reference.

- **The generation of ellipsoid models via PyMOL [68] was enabled based on structure refinement with TLS parameters through PHENIX [69].**

12. l. 495. what is a Kirchhoff matrix? ✓✓✓✓

It is basically the contact map that we define at the start of a Gaussian Network Model (GNM) analysis. It is an NxN symmetric matrix in which N is the atom number. The Hamiltonian potential calculated for GNM requires the Kirchoff matrix to be defined. In this study we used the python module ProDy for doing so; we simply defined the Kirchhoff matrices with the selected atom coordinates, selected a cut-off distance (7.3 Å) to assume pairwise interactions, and used the default spring constant of 1.0 for both our structures. We decided to add the information about the spring constant to the methods section as follows: **“Default spring constant of 1.0 was used for both structures.”**

13. Fig. 3. Poor expression in the figure caption, please correct ✓✓✓✓

Thank you Reviewer #1 for the comment. Changed "Superposition of the binding site of APO_SFX and selenobiotin bound (PDB ID:5JD2) structures of streptavidin." to **"Superposition of the biotin-binding sites for each chain of the Apo-SFX and Holo-SFX structures."**

14. Fig. 5 Poor expression in the figure caption, please correct. ✓✓✓✓

Thank you Reviewer #1 for the comment. Changed "Binding site interactions of APO_SFX structure of streptavidin for each chain" to **"Representation of coordinated water molecules and polar interactions near the binding sites for each chain of Apo-SFX structure."**

15. Fig. 6. Delete 'based on B-factor' ✓✓✓✓

Thank you Reviewer #1 for the comment. Deleted 'based on B-factor' phrase in Fig. 6

****
****

Reviewer #2 (Remarks to the Author):

The changes the authors made to the manuscript improved both reading and clarity. I appreciated the clear answers in the rebuttal letter for all the different concerns raised by the reviewers. The introduction on GNM analysis is much better. The overall explanation the authors gave about the necessity for Apo-SFX structure is satisfactory.
The second version of the manuscript is much better.

I still have few minor remarks:
1) I disagree with part of the answer in the rebuttal letter from my minor remark n°5. I was just curious about the criteria applied with CrystFEL to determine the resolution cut-off. I understand it is based on CC* which is quite specific for CrystFEL and XDS. I disagree with the sentence "Resolution cut-off for all crystallography data is generally based on CC* and not CC1/2." Different programs use different parameters, e.g. Dials uses CC1/2. Also, CC* is based on CC1/2. ✓✓✓✓

We sincerely apologize for our oversight of including the following sentence ***"Resolution cut-off for all crystallography data is generally based on CC* and not CC1/2."*** We fully agree with our reviewer that different programs use different parameters for resolution cutoff determination.

2) Line 77: I don't understand the analogy to "plug and play" type of molecule ✓✓✓✓

The term "plug and play" is expanded to "**Streptavidin is a molecule characterized by its interactions with a wide range of biotinylated molecules and is widely used in biotechnology. Its binding with biotinylated molecules is diffusion-limited and has a remarkably high binding**

efficiency. Streptavidin can form new nano-assemblies by using biotinylated non-biological building blocks as well as organic molecules such as sugar, protein, and nucleic acids [15].”

3) Line 92-94: I agree with the authors about the fact that “cryogenic temperature can introduce bias”. Could the author backup this statement with reference(s)? ✓✓✓✓

The relevant text has been edited and the reference has been added to the relevant text as follows: **“... In addition, cryogenic temperature may perturb the overall protein backbone fold [26].”**

4) Lines 114, 280, 314, 361, 362, sup Fig 2 and 6…: Could you please either call in the manuscript the loop L3,4 or L3/4 loop? ✓✓✓✓

We would like to thank Reviewer #2 for his/her comments and apologize for this mistake. The indicated loop is referred to as **L3/4** throughout the main text for the consistency.

5) Line 141: I don’t understand “R-free 0.253 without R-free…” ✓✓✓

R-work (writing mistake it should be R-work in the mentioning sentence in line 141 of main text (Result section)) was not indicated in the PDB databank for the ambient temperature synchrotron apo-structure of streptavidin (PDB ID: 1SWB). The sentence is corrected as **“1SWB was determined at 1.85 Å resolution with an R_{work} of 0.17 and R_{free} of 0.25 while the Apo-SFX structure was determined at 1.7 Å resolution with an R_{work} of 0.19 and R_{free} of 0.22.”**

6) Line 225: what is the cut-off distance? I suppose it is the cut-off for the correlation between residues. Why do you have 2 different cut-off distances? What is the unit? Å? ✓✓✓✓

We mention in the Materials & Methods section that only 1 cut-off was used for consistency actually: “Same cutoff distance of 7.3 Å was selected in both models to assume pairwise interactions.”. However, we realized upon the reviewer’s comment that the sentence at line 225 appears to misguide the reader and tried to fix it as follows: **“They showed high correlation at the selected cut-off distance of 7.3 Å: overall correlation with B-factors was 0.785 in the GNM of Holo-SFX structure and 0.646 in the GNM of Apo-SFX structure. ”**

7) Line 307-309: Just being curious… You should have enough structural data to build an hybrid tetramer with each dimer having only one biotin bound. GNM studies on this tetramer may answer your hypothesis. Maybe for another paper… ✓✓✓✓

It is a terrific suggestion, we would like to thank the reviewer for this. Indeed, such a study could be done and provide additional data regarding the cooperative allostery mechanism we have suggested.

8) Line 458: Replace (by 28% ✓✓✓✓

The sentence is replaced as **"For streptavidin crystals, the final dataset included 384,250 hits with a total of 106,021 indexed patterns (28%) was merged into a final dataset ($P12_11$, unit cell: a = 47.40 Å, b = 87.70 Å, c = 58.90 Å; α = 90.00, β = 98.90, γ = 90.00) (Supplementary Table 1)."**

9) Line 465: I would add here the answer you gave me for my remark n°18 about the low completeness (~80%) in the high-resolution shell for the cryo structure. ✓✓✓

We would like to thank reviewer 2 for his/her comment. The mentioned sentence was added to line 465. The sentence is indicated below.

- **The resolution cutoff set to 1.1 Å without negatively impacting R_{free} and R_{work} (Supplementary Table 1)**

10) Line 469: as you put the instrument used at LCLS, I would add the instrument BL-12-2 as well for SSRL. ✓✓✓✓

The sentence is corrected with additional information as follows:.

- **For cryo-synchrotron structure, X-ray diffraction data ($P12_11$, unit cell: a = 46.36 Å, b = 85.76 Å, c = 58.13 Å; α = 90.00, β = 98.71, γ = 90.00),were collected by using a Dectris Pilatus 6M detector installed at BL12-2 instrument at SSRL, which was processed with *XDS* [62] package for indexing and scaled by using *XSCALE* [62].**

11) Figure 4E: I don't really understand which one is 5JD2 and which one is Apo-SFX. In blue, you have close conformation so it should be 5JD2 and in red open conformation then Apo-SFX. But chain A from Apo-SFX is in closed conformation, hence my confusion for the Chain A in figure 4E. ✓✓✓✓

We believe our Reviewer 2 refers to Figure 8E and we first apologize for the confusion caused with the labels. To clarify, the labels in legends were changed from open to Apo-SFX (red) and from closed to 5JD2 (blue) for consistency which can be seen in the following figure. Although the conformations should be similar in chain A for both structures, water molecules in Apo-SFX structure are the main reason for similarity but they were not included in the GNM analysis. Selenobiotin fills the place of the waters in the Holo-SFX (5JD2) and we included atoms of this ligand in the analysis to see precisely the effect of the ligand. We think that is the reason for the fluctuation differences at binding sites between them. The following sentences were also added to the main text to clarify this issue:

- Results: **“Additionally, although the conformations should be similar in Apo-SFX chain A for both structures, fluctuation differences were observed between them at binding sites.”**
- Discussion:**”One reason for the striking difference in fluctuations is that selenobiotin was included in the GNM of Holo-SFX (5JD2) and, therefore, the ligand’s effect on decreasing the flexibility of the region was more clear in the protein’s global motions.”**

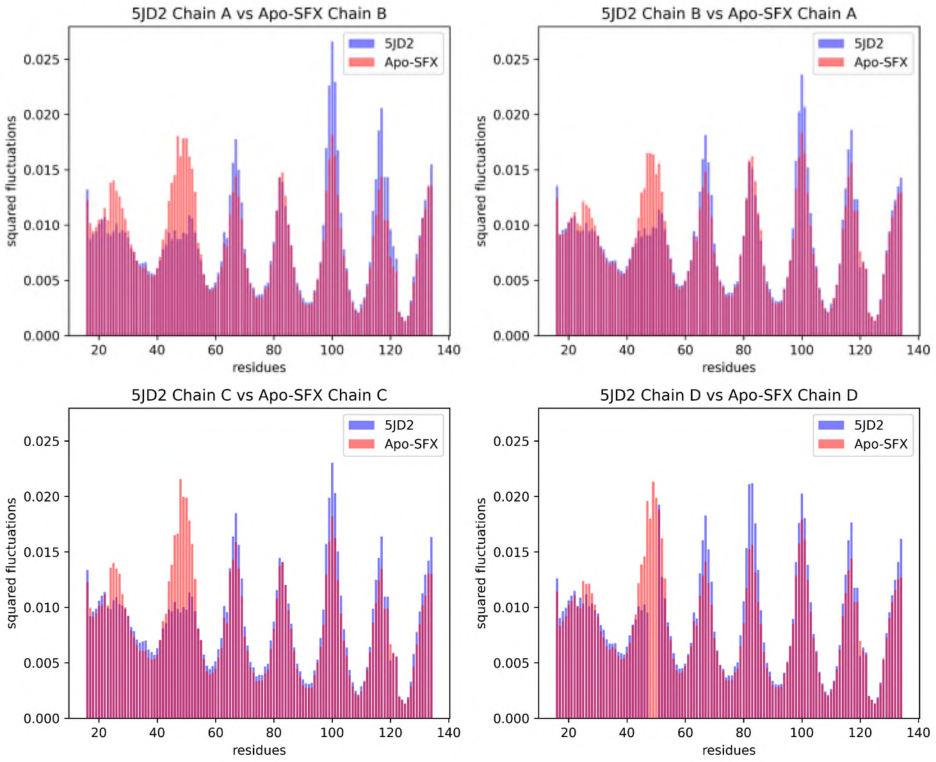

Figure: The legends were changed from open to Apo-SFX (red) and from closed to 5JD2 (blue) for consistency.

12) Supplementary Fig. 1: What is the difference between green arrow and red dot? And what is the red arrow? ✓✓✓✓

The sentence is corrected with additional information as follows: **“Red dots represent residues in contact with ligand (biotin), while inverted triangles that are colored in green and red represent functional residues of repeats.“**

13) Supplementary Fig. 5: Yellow text is hard to read ✓✓✓✓

Yellow text of Supplementary Fig. 7 (updated version) is replaced with a darker tone to increase the contrast.

14) Supplementary Fig. 11: Black square are not described in the legend ✓✓✓✓

The explanation is added to the legend of Supplementary Fig. 13 (updated version). **"The binding pocket for selenobiotin which is colored in light pink is indicated with black squares in the panels."**

15) Supplementary Table 1: replace LCLS by LCLS (MFX) and you could change Beamline by Instrument if you like. ✓✓✓✓

Thank you for the comment of reviewer #2. The sentence is changed as below.

- **Apo-streptavidin structures, obtained at LCLS (MFX) and cryo-synchrotron at SSRL (BL12-2), were determined by using the automated molecular replacement program *PHASER* [65] implemented in *PHENIX* software [66] with the previously published Holo-SFX structure as a search model and initial rigid-body refinement [43]**

16) Supplementary Table 1: I am still missing Rmeas for the cryo structure. ✓

This was a typo on the table and we thanks our Reviewer 2 for bringing this to our attention

****

Reviewer #3 (Remarks to the Author):

The manuscript by Ayan et al. has **increased in clarity from the previous version**. Most concerns have been addressed. The novel Apo-SFX structure provides enhanced details of the tetrameric streptavidin, several segments and side chains are resolved continuously. Particularly, the 3,4 loop (L3,4) of each monomer is clearly resolved. The authors use GNM to explore the correlated movements of residues on each monomer as part of the Apo-SFX and compared to the biotin bound structure, also solved at ambient temperatures (PDB: 5JD2). However, the correlation between residues in L3,4 and others in the binding site, dimer interface, or dimer/dimer interaction appear to be unnoticed by GNM or they are absent. This, in turn, would indicate that the "Lid" movement does not propagate to other parts of the complex or it is not influenced by the rest of the complex. Nevertheless, the Apo-SFX structure shows that L3,4 is closed in one monomer out of three. The opposite occurs in the Holo-SFX structure, where one monomer shows an open lid albeit being bound to biotin. The authors and others interpret these findings as L3,4 being intrinsically flexible. However, this can be also explained by coordinated allosterism. In this case, the GNM should have evidenced such correlations.

Altogether, the work by the DeMirci group provides new insights into the functioning of the multimeric streptavidin.

Here, you can find several issues that were annotated during this revision.

Minor issues:

1. Revise the order of supplementary figures. They do not appear orderly in the text. ✓✓✓

We thank our Reviewer 3 for bringing this to our attention. Revised the order of supplementary figures considering this comment.

2. Lines 65-67: “L3,4…. Is the crucial part of streptavidin that interacts with biotin and regulates the binding”. References required. However, whether L3,4 loop regulates biotin binding or not may be overstated. Please, revise also the following sentence, appears redundant. ✓✓✓✓

Thank you Reviewer #3 for the comment. Lines 65-67 have been modified by adding reference as **“ Apo-state streptavidin loop (L3/4; residues 45–52) (Supplementary Fig. 1) is the crucial segment of streptavidin that interacts with biotin and regulates binding action [10].”** In addition, the following sentence was deleted: **"The presence of a flexible loop L3/4, surrounding the biotin and connecting β-strands (β3/4), plays an important role in the binding interaction [10]. "**

3. Lines 92-94: “ However, the available apo-state structures are still limited as cryogenic temperatures can introduce bias for structure determination by disrupting the overall protein backbone fold.”
Review this sentence. First, there are apo-state structures available. Second, what is the evidence available for structural biases introduced by cryogenic temperatures?? Need some references here. ✓✓✓✓

We would like to thank Reviewer #3 for this comment. Changed “However, the available apo-state structures are still limited as cryogenic temperatures can introduce bias for structure determination by disrupting the overall protein backbone fold.” to **“There are existing cryogenic apo-state structures in the literature [24]; however, temperature artifacts could introduce errors, preventing successful computational predictions [25]. In addition, cryogenic temperature may perturb the overall protein backbone fold [26]. Therefore, studies have recommended greater caution when referring only to cryogenic structures [24, 25].”**

4. Line 108: “Apo_SFX” vs “Apo-SFX”. Correct along the MS for consistency. Also, I suggest to use Biotin-SFX or Holo-SFX instead of (5JD2) for clarity. ✓✓✓✓

Changed “Apo_SFX” to “Apo-SFX” and changed “5JD2” to “Holo-SFX”.

5. Lines 139-140: “First we compared the ambient temperature synchrotron apo-structure of streptavidin (1SWB) with our Apo_SFX structure”
Reference is required for PDB 1SWB. ✓✓✓✓

Thanks to Commenter #3 for the comment. Reference added to main text for PDB 1SWB as follows **"First we compared the ambient temperature synchrotron apo-structure of streptavidin (1SWB) with our Apo-SFX structure [30]."**

6. Line 147: a reference for 3RY1 is required. ✓✓✓✓

Reference added to main text for PDB 3RY1 as follows **"Moreover for a fair comparison, we compared the electron density of binding site residues between our Apo-SFX structure and the latest synchrotron cryo-structure of apo-streptavidin (PDB ID: 3RY1) [31]..."**

7. Line 150: PDB 7EK8? ✓✓✓✓

PDB 7EK8 refers to our crystal structure of apo streptavidin at ambient temperature in the current manuscript. This was revised as **"Apo-SFX"**

8. Lines 171-173: "…..biotin substrate. Interestingly, this conformational change is more prominent for chains B, C, and D."
This sentence appears unclear, does it relate to Apo_SFX or to 6J6K?. Refer to a figure that shows these conformational changes. ✓✓✓✓

Supplementary Figure 9 was assigned for referring to the indicated conformational changes as follows **"Conformational differences of L3/4 indicate the non-symmetric binding of biotin with monomers (Supplementary Fig 9)."**

9. Line 183: review the use of "determined", likely you mean "found" ✓✓✓✓

Thanks to Reviewer #3 for the comments. Changed "As expected, the conformational changes were determined in the binding pocket." to **"As expected, conformational changes were found in the binding pocket."**

10. Line 280: "3/4 loop" should read ✓✓✓

Thanks to Reviewer #3 for the comment. The related sentence was fixed as **"It must be noted that chain D of the Holo-SFX structure was observed with disordered residues at L3/4 (Fig. 3D) which also can be clearly seen from the GNM analysis graph (Fig. 8E)."**
